# Real-Time Wood Chemotyping Using a Low-Cost and Compact Mass Spectrometer

**DOI:** 10.1021/acsomega.5c13161

**Published:** 2026-03-05

**Authors:** Thays V. C. Monteiro, Mariana Fioramonte, Renan Pirolla, Alexandre Bahia Gontijo, Cristiano S. Nascimento, Niro Higuchi, Mário Augusto Gonçalves Jardim, Maíra Fasciotti

**Affiliations:** 1 Graduate Program in Metrology and Technology, Laboratory of Organic Analysis, National Institute of Metrology, Quality and Technology (INMETRO), Duque de Caxias 25250-020, Brazil; 2 Waters Technologies Brazil, São Paulo 06455-020, Brazil; 3 Forest Products Laboratory/Brazilian Forest Service (LPF/SFB), Brasília 70818-900, Brazil; 4 Institute of Biological Sciences, Graduate Program in Ecology (UnB/ICB/PPGECL), University of Brasília, Brasília 70910-900, Brazil; 5 Forest Management Laboratory/National Institute for Amazon Research (LMF/INPA), Manaus 69060-062, Brazil; 6 Museum Paraense Emílio Goeldi/Coordination of Botany (MPEG/COBOT), Belém 66040-170, Brazil

## Abstract

The ongoing deforestation of critical ecosystems such as the Amazon—driven largely by illegal logging—highlights the urgent need for accessible, scientifically robust tools for reliable timber identification. Traditional anatomical methods often fall short when distinguishing between morphologically similar species, while advanced analytical techniques, though accurate, are typically expensive, laboratory-bound, and impractical for massive timber trade monitoring. In this study, we report the first application of a compact, low-cost mass spectrometer for direct chemotyping of tropical hardwoods, aiming to explore its potential application in timber identification in regulatory and enforcement contexts. Four high-value species prone to fraudulent substitution—*Swietenia macrophylla* (mahogany), *Carapa guianensis* (andiroba), *Cedrela odorata* (cedar), and *Hymenaea courbaril* (jatoba)—were analyzed from methanol/water (3:1) extracts using atmospheric solids analysis probe mass spectrometry (ASAP-MS), an ambient ion source, and a single quadrupole mass analyzer (RADIAN ASAP, single quadrupole), without chromatographic separation. Chemometric analysis via PCA–LDA (combination between principal component analysis and linear discriminant analysis) revealed clear species-specific chemical fingerprints and achieved 100% classification accuracy with minimal sample preparation. The proposed method combines high discriminatory power, lower cost, and operational simplicity in a laboratory-independent configuration with potential applicability across enforcement contexts such as airports, seaports, or mobile units. Its minimal infrastructure requirements suggest potential suitability for future on-site applications, contributing to improved analytical support in timber trade enforcement operations. This study provides a proof of concept for decentralized analytical approaches that may contribute to improved timber trade monitoring and biodiversity protection.

## Graphical abstract

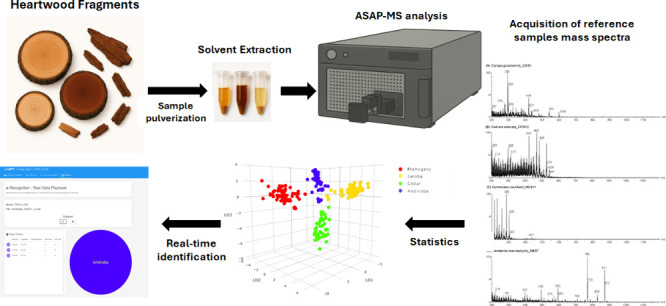

## Introduction

Deforestation and illegal logging in tropical biomes such as the Amazon remain some of the most pressing global environmental challenges. Since 1988, Brazil’s Legal Amazon has lost over 497,000 km^2^ of forest cover, with 6288 km^2^ cleared in 2024 alone.^1^ Beyond forest cover loss, deforestation causes widespread ecological disruption and increases fire incidence.^2,3^

IBAMA (Brazilian Institute of Environment and Renewable Natural Resources) and MMA (Ministry of the Environment and Climate Change) estimate that 30–50% of harvested timber in Brazil originates from unauthorized or fraudulent sources, frequently involving species misidentification or falsified origin.^4–6^ Despite advances in satellite monitoring and the expansion of protected areas, enforcement gaps continue to facilitate illegal timber harvesting.^1,7^

In biodiversity-rich countries like Brazil, a persistent challenge is the identification of processed wood materials, often commercialized without leaves, flowers, or fruits and therefore lacking visible diagnostic botanical features. Accurate species identification is essential for ensuring transparency and legality in the timber trade as endangered or protected species may otherwise be fraudulently marketed as legally permitted taxa.

Current timber chain-of-custody systems rely largely on self-declared documentation, making them vulnerable to fraud, particularly through intentional species misidentification and the laundering of illegally harvested timber.^8–10^ Although anatomical analysis remains widely used,^11^ it often resolves only to the genus level and may fail for closely related taxa, even when performed by trained anatomists.^12–14^

Beyond its role in combating illegal logging, reliable wood species identification also supports sustainable forest management by ensuring compliance with approved harvesting plans and improving transparency across legal timber supply chains. In this context, rapid and objective species authentication tools can benefit both enforcement actions and proper management of legally harvested forests.

The species investigated in this study include *Swietenia macrophylla* (mahogany) and *Cedrela odorata* (cedar), both listed in Appendix II of CITES and subject to strict trade controls, as well as *Carapa guianensis* (andiroba) and *Hymenaea courbaril* (jatoba), which, although not CITES-listed, are high-value timbers frequently involved in commercial substitutions. The unequivocal discrimination among these species is therefore essential for effective enforcement and prevention of the laundering of protected timber through legal supply chains.

Against this background, analytical approaches capable of providing rapid and objective species discrimination have gained increasing relevance. Wood chemotyping, based on the analysis of secondary metabolite profiles (extractives), offers a powerful alternative for species differentiation and identification and has also the potential for geographic differentiation.^15–17^ These chemical markers enable precise discrimination, even among morphologically similar woods.^18^

The structural diversity of plant metabolites poses analytical challenges and has motivated the application of multiple analytical techniques for wood characterization, including near-infrared spectroscopy (NIRS), nuclear magnetic resonance (NMR), and mass spectrometry (MS).^19–24^ While spectroscopic methods are attractive for rapid screening, limited selectivity may hinder discrimination in chemically complex samples. NIRS has been widely applied for rapid species and origin screening, but its performance decreases in cases involving closely related taxa or complex chemical matrices.^19,20,25,26^ Nuclear magnetic resonance (NMR) provides detailed structural elucidation for chemical differentiation between species, despite being costly and complex.^21,22,27^

Other approaches have been applied to wood identification and traceability, including DNA-based methods (e.g., DNA barcoding and fingerprinting), which show promise^28–31^ but face limitations in distinguishing morphologically similar species^32^ and in geographic differentiation due to genetic variation.^33,34^

Isotope ratio mass spectrometry (IRMS) has been employed to differentiate geographic origin through the isotopic composition of elements such as C, N, O, H, and Sr,^35–42^ although its resolution decreases in environmentally homogeneous regions.

Chromatographic techniques, such as GC-MS (gas chromatography coupled to mass spectrometry), have been employed in the chemical profiling of wood. However, GC is limited to volatile organic compounds (VOCs), which often do not provide robust and definitive chemical profiles to characterize specific species.^43–45^

Mass spectrometry (MS) has emerged as a powerful tool for wood species identification through the analysis of secondary metabolites,^24,43,45,46^ particularly using ambient and direct analysis approaches. Techniques such as DART-TOFMS (direct analysis in real time–time-of-flight mass spectrometry), adopted by the U.S. Forest Service, provide rapid and accurate species discrimination and are widely applied in forensic and chemotaxonomic contexts.^11,47–53^ Similarly, direct infusion ESI-MS (electrospray ionization mass spectrometry) approaches such as V-EASI-MS (Venturi easy ambient sonic-spray ionization mass spectrometry) have differentiated native and plantation-grown mahogany (Brazilian and African) species in Brazil.^54–56^ These MS-based approaches eliminate chromatographic separation and generate species-specific spectral fingerprints within minutes with the potential for interlaboratory reproducibility. In contrast, chromatography-coupled high-resolution platforms, such as LC-HRMS (liquid chromatography–high-resolution mass spectrometry)^57,58^ and using ESI-QTOF (electrospray ionization quadrupole time-of-flight) mass spectrometers, offer enhanced chemical detail and accurate compound identification, albeit with increased operational complexity and time.^59,60^

Despite the strong analytical performance of MS-based approaches, their routine use in enforcement is limited by high instrument costs, infrastructure demands, and technical expertise requirements. There is, therefore, a need for cost-effective, field-ready MS solutions capable of operating outside traditional laboratory environments.

Here, we present the first report of a chemotyping method based on the RADIAN ASAP mass spectrometer, a compact, low-cost instrument equipped with an atmospheric solids analysis probe (ASAP) ion source, and a single quadrupole analyzer.

The method enabled accurate discrimination of similar and commercially valuable timber species (*Carapa guianensis*, *Cedrela odorata*, *Hymenaea courbaril*, and *Swietenia macrophylla*), including CITES Appendix II-listed taxa, using chemometric analysis of mass spectral profiles. The selection of the four studied species was designed as a proof of concept to evaluate the analytical robustness of the RADIAN ASAP platform when it is applied to complex tropical wood matrices. Rather than addressing a specific taxonomic ambiguity, this study aimed to assess whether reproducible chemical fingerprints could be generated and reliably classified using a compact and low-cost mass spectrometer.

This approach may reflect enforcement scenarios in which analytical tools accessible to nonspecialists may be beneficial. Owing to its simplicity and minimal training requirements, the proposed approach demonstrates potential for deployment at ports, airports, and law-enforcement checkpoints, without the need for controlled laboratory conditions typically required by high-resolution MS platforms.

## Methods and Materials

### Samples

Fragments of wood samples were obtained through collaborations with Brazilian xylotheques, with priority given to four timber species that exhibit visual similarities when observed with the naked eyes in the field.^61,62^ To ensure accurate taxonomic verification and facilitate the comparative analysis of diagnostic features, the collaborating xylotheques provided high-resolution macroscopic images of the transverse and longitudinal surfaces for each accession by employing distinct imaging protocols. Transverse surfaces were prepared using standard wood anatomy protocols, including polishing with progressive grit sandpaper (up to 600 grit) and acquired using stereomicroscopes at 20× magnification. This specific magnification allowed for the detailed visualization of cellular structures, such as vessel arrangement and parenchyma patterns. In contrast, longitudinal surfaces were imaged without magnification (macroscopic view) to replicate naked-eye observations, focusing on general wood appearance, grain patterns, and texture, which are critical for initial field identification. The species analyzed in this study were *Carapa guianensis*, *Cedrela odorata*, *Hymenaea courbaril*, and *Swietenia macrophylla*. A total of 60 heartwood samples were examined, collected from various locations, predominantly in the states of Amazonas and Pará in the Brazilian Amazon (Table 1). The complete data set of the samples analyzed in this study is provided in the Supporting Information (Table S1).

**Table 1:** Woods Selected for the Study, Number of Samples, and Collection Site

family	scientific name/popular	no. of samples	collection location
Meliaceae	*Carapa guianensis* Aubl. (andiroba)	18	Brazil (Amazonas; Pará) and Venezuela
	*Cedrela odorata* L. (cedar)	15	Brazil (Amazonas; Pará; Rondônia)
	*Swietenia macrophylla* King (mahogany)	16	Brazil (Amazonas; Pará; Rondônia)
Fabaceae	*Hymenaea courbaril* L. (jatoba)	11	Brazil (Amazonas; Mato Grosso; Pará) and Suriname

### Sample Preparation

Two distinct approaches were evaluated for sample preparation during the development of the analytical method: direct analysis of the pulverized solid wood and analysis of extracts obtained through extraction using a methanol/water solution in a 3:1 ratio. For the latter, a small amount of sample (3–5 mg) was transferred to a polypropylene microtube, to which 100 μL of the extraction solvent was added. The microtubes were then vortexed for approximately 30 s. The capillary tube from the ASAP source was subsequently immersed in the solvent, directly coupled, and inserted into the ASAP source. For the analysis of pulverized solid wood, the capillary tube was inserted into the wood powder so that a small quantity of particles adhered to it, which was then introduced directly into the ASAP source.

### Method Optimization

Mass spectral data were acquired using a gas temperature ramp in which the temperature was incremented by 100 °C every minute, varying from 100 to 600 °C, during 6 min, at a 2 Hz scan rate. Data were acquired using 5 μA on corona (current mode), a 120 °C source temperature, and two different cone voltages, 35 and 40 V.

### Data Acquisition

Data were acquired on a RADIAN ASAP mass spectrometer (Waters Co.). ASAP (atmospheric solids analysis probe) ionization was performed using positive mode, 5 μA on corona (current mode), a 120 °C source temperature, and a 400 °C gas heater temperature, using nitrogen as a gas. Data were acquired from *m*/*z* 200 to 1200, with a 2 Hz scan rate and a cone voltage of 35 V, for 1 min. Samples were injected in triplicate in a random order, injecting a sample pool every 10 samples. Prior to all sample triplicates, a blank consisting of a clean capillary inserted in a microtube with methanol:water (a “blank”) was injected.

### Statistics

The combination of principal component analysis (PCA) and linear discriminant analysis (LDA) was employed for the classification of the samples based on their characteristic spectral profiles. The parameters used for this model were as follows: 60 PCA components, 3 linear discriminants (required due to the presence of four groups to be separated), an outlier threshold based on a standard deviation criterion set at 5 standard deviations, and an *m*/*z* binning resolution of 0.1 Da, using the full spectral range of *m*/*z* 200–1200.

The employed model was validated by assessing its performance using the same data on which it was trained. The training data were used as surrogate test data points. A portion of the training set was withheld from the model, and a reduced-size model was constructed. Validation was performed using a stratified 5-fold cross-validation method (20% holdout).

For the model recognition test, three samples from each species were randomly selected and excluded from the training set (CGS5, CGS11, CGS17, COS4, COS7, COS13, HCS6, HCS9, HCS11, SMS3, SMS5, and SMS15, as listed in Table S1 of the Supporting Information). These samples were later introduced into the model for classification, simulating a real-world scenario of unknown-sample identification.

## Results and Discussion

### Method Optimization

In this study, we describe the development and application of a rapid method for the identification of wood species based on their chemical profiles using a RADIAN ASAP mass spectrometer (Waters Corp., Milford, US). This instrument is a single quadrupole mass spectrometer equipped with an ASAP ion source, an ambient ionization source that allows for the direct introduction of solid, volatile, or semivolatile liquid samples into the mass spectrometer without the need for prior preparation or chromatographic separation. The operating principle is based on the thermal volatilization of the sample followed by ionization via corona discharge under conditions similar to atmospheric pressure chemical ionization (APCI). The sample is applied to a glass capillary (probe), which is inserted into the ion source and rapidly heated to high temperatures (up to approximately 600 °C), promoting the volatilization of the compounds present in the sample. The resulting vapors pass through an electrical discharge region, where ionization occurs via reactions with ions formed from the solvent or ambient air. The generated ions are then directed into the mass analyzer. This technique is particularly useful for the analysis of organic compounds in various matrices such as pharmaceuticals, paints, fibers, food products, and natural products, enabling efficient analyses with minimal analytical intervention.^63–66^

Since the instrument allows for the analysis of both solid and liquid samples, the first stage of the study focused on evaluating sample preparation methods. Both the direct analysis of pulverized solid wood (results not shown) and the analysis of extracts obtained using a methanol/water solution in a 3:1 ratio were assessed. Both approaches proved feasible for application with the employed technique, generating similar and reproducible spectra (results not shown). Ideally, direct analysis of the wood without any sample preparation would simplify the analytical process. However, it was observed that direct analysis of solid samples led to greater contamination of the mass spectrometer and a rapid loss of sensitivity, which was not the case with the methanol:water extracts. This is plausible given that wood is primarily composed of nonvolatile and nonionizable long-chain polymers such as lignin and cellulose, while the extractive fraction, responsible for secondary metabolite content, represents only about 5–10% of the wood’s chemical composition.^67^ Therefore, it is expected that analysis of the extract, free from cellulose and lignin, would result in lower deposition of nonvolatile components in the ion source, thereby improving analytical sensitivity. Considering these findings, subsequent analyses were performed using only the methanol/water extracts and also considering that the extraction method is simple and time-efficient. It is important to note that the use of solvent extracts does not introduce additional methodological complexity; rather, it improves spectral representativeness and reproducibility by enabling the analysis of larger and more homogeneous sample masses while simultaneously reducing ion source contamination during extended analytical sequences. Operationally, the extraction step consists solely of immersing wood fragments in a solvent, representing a minimal and rapid preparation step when weighed against the gains in spectral stability and instrument robustness.

Also, in this study, a precise and fixed mass was intentionally not employed. Instead, a small range (3–5 mg) was selected to evaluate method robustness under less controlled sampling conditions. Our aim was to evaluate whether the method remains robust under these practical circumstances, reflecting practical sampling variability. Even with this slight variability in sample mass, the analytical performance and classification results remained consistent, supporting the method’s reliability.

The subsequent stage focused on optimizing the method for spectrum acquisition, specifically evaluating the nitrogen gas temperature in the ion source and cone voltage. These ionization and desorption parameters were optimized using a qualitative, univariate approach, in which each instrumental condition was varied independently and selected based on signal intensity, spectral stability, and minimization of excessive fragmentation, following standard practices in mass spectrometry-based method development. Regarding temperature optimization, Figure 1 presents the total ion currents (TIC) obtained under varying conditions. An increase in the TIC is observed as the gas temperature increases.

**Figure 1:**
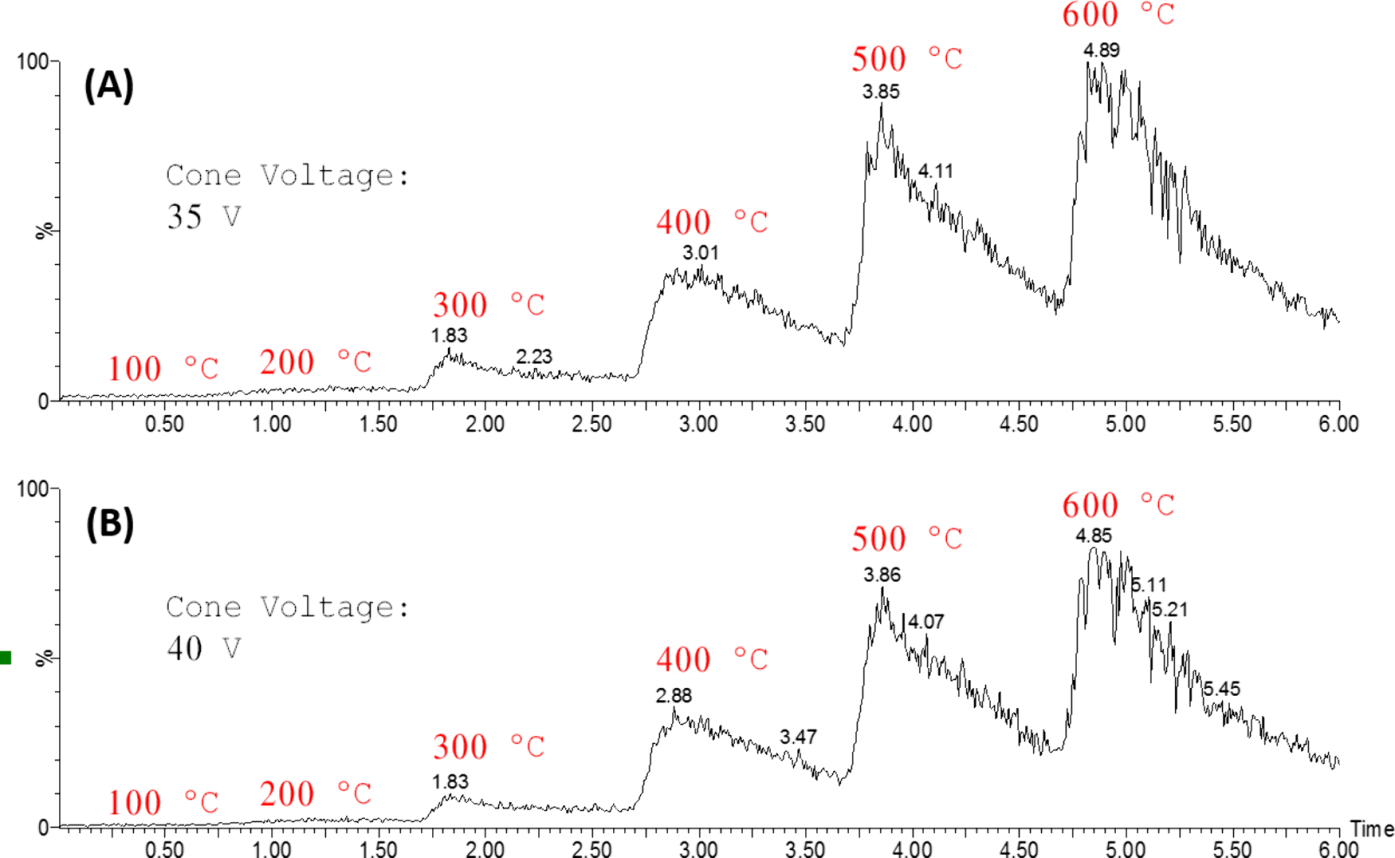
Total ion currents (TIC) obtained under method optimization employing a gas temperature ramp as represented in the pictures. (a) Using 35 V on cone voltage and (b) 40 V on cone voltage.

Figure 2 illustrates the comparison of mass spectra acquired for a representative mahogany sample at different gas temperatures using a fixed cone voltage of 35 V. As the temperature rises, there is a notable enhancement in the intensity of ions with higher *m*/*z* values, such as *m*/*z* 593, *m*/*z* 769, and *m*/*z* 871, up to a gas temperature of 400 °C. At 500 °C, however, the intensities of these ions begin to decline, suggesting potential fragmentation in the ion source or compound degradation. Based on these observations, 400 °C was selected as the optimal gas temperature for subsequent analyses. Figure 3 depicts the comparison between cone voltages of 35 and 40 V. As shown, an increase to 40 V results in signs of in-source fragmentation (ISF). Therefore, a cone voltage of 35 V was chosen for data acquisition to minimize fragmentation and preserve the analyte integrity.

**Figure 2:**
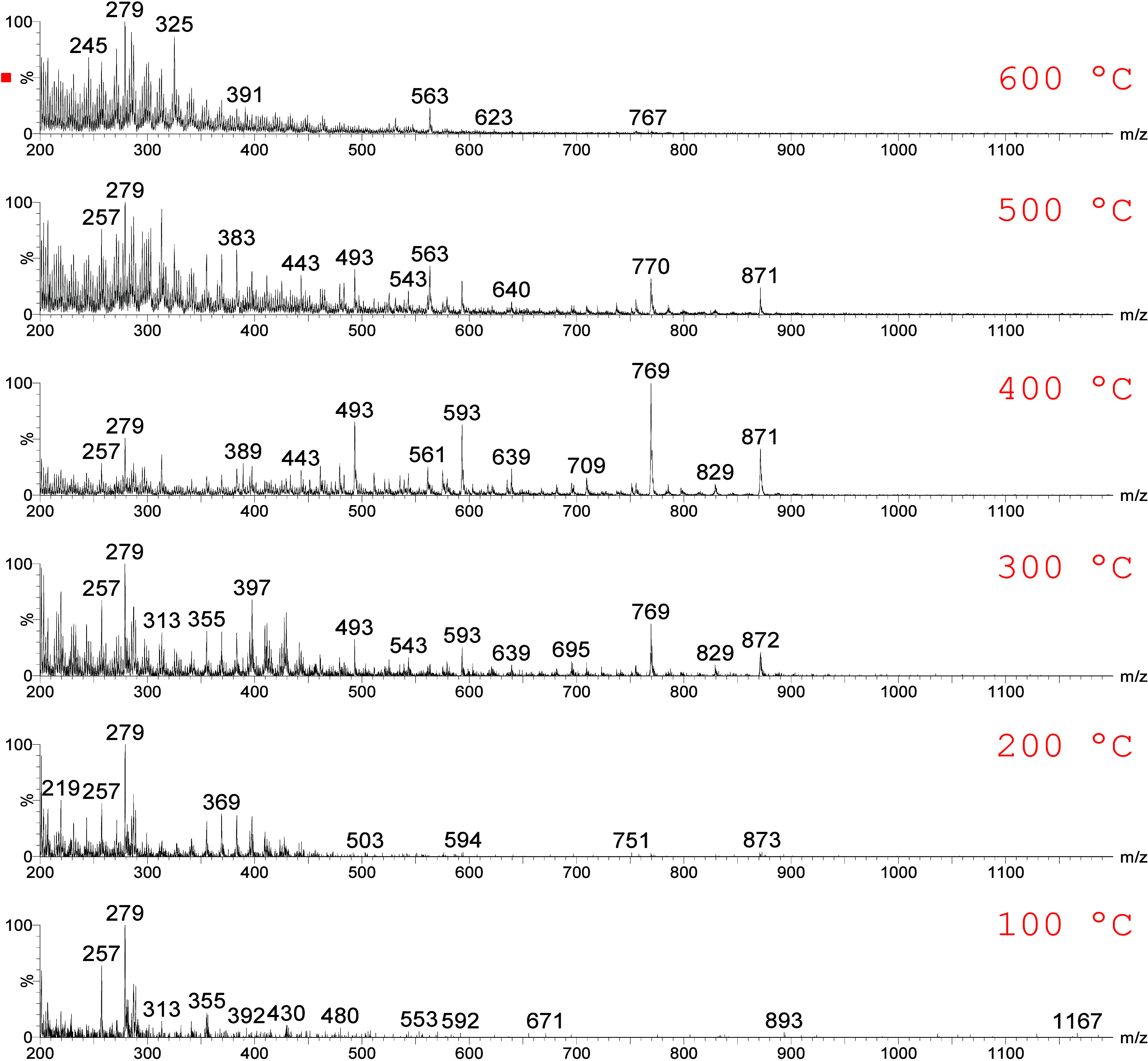
Full-scan mass spectra obtained using 35 V on cone voltage at different temperatures for a representative mahogany sample.

**Figure 3:**
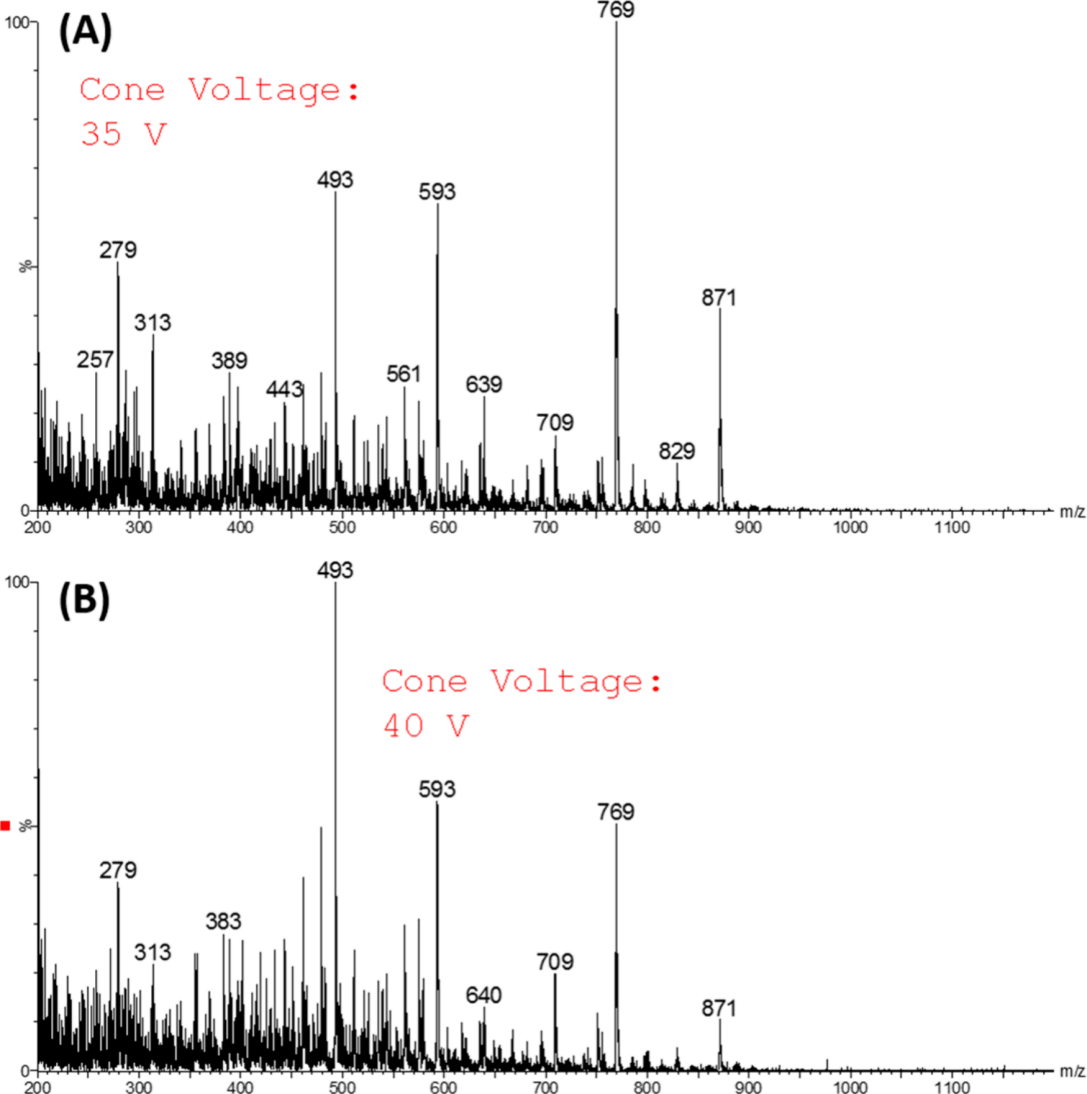
Full-scan mass spectra obtained (mahogany sample) using 400 °C of gas temperature at (a) 35 and (B) 40 V on cone voltage.

After completing the optimization steps, the final analytical method was established based on the analysis of extracts obtained from pulverized samples using a methanol/water solution (3:1, v/v). All spectra were acquired by using a cone voltage of 35 V and a desorption gas temperature of 400 °C, the conditions that provided the most informative and stable spectral profiles. These optimized parameters were subsequently applied to all samples analyzed in this study.

From an initial analysis, a comparison of the representative spectra obtained for the four wood species, andiroba, cedar, jatoba, and mahogany (Figure 4), reveals clear differences among them, with distinct characteristic ions for each sample. The andiroba sample (Figure 4A) exhibits a distribution of ions primarily in the *m*/*z* range of 270–440, while cedar (Figure 4B) shows characteristic ions in the *m*/*z* range of 400–500. The jatoba sample (Figure 4C) displays its most intense ions concentrated in a narrower range between *m*/*z* 200 and 300. Finally, the mahogany sample (Figure 4D) presents ions of higher *m*/*z* values, predominantly around *m*/*z* 700. These results suggest that each of the four wood species possesses a characteristic spectral profile, supporting the feasibility of applying multivariate statistical methods to develop a robust classification model capable of efficiently distinguishing between the analyzed species. Table 2 provides a summary of the five most intense ions identified in each representative spectrum shown in Figure 4. As RADIAN ASAP is a low-resolution mass spectrometer, no inference regarding the ion’s identity must be performed, especially considering that there is no previous separation of the compounds (i.e., by chromatography) being performed. Additionally, the Supporting Information includes spectra from all analyzed samples, demonstrating that different species, even when sourced from varying geographic regions, tend to exhibit highly characteristic spectral patterns, with only rare exceptions.

**Figure 4:**
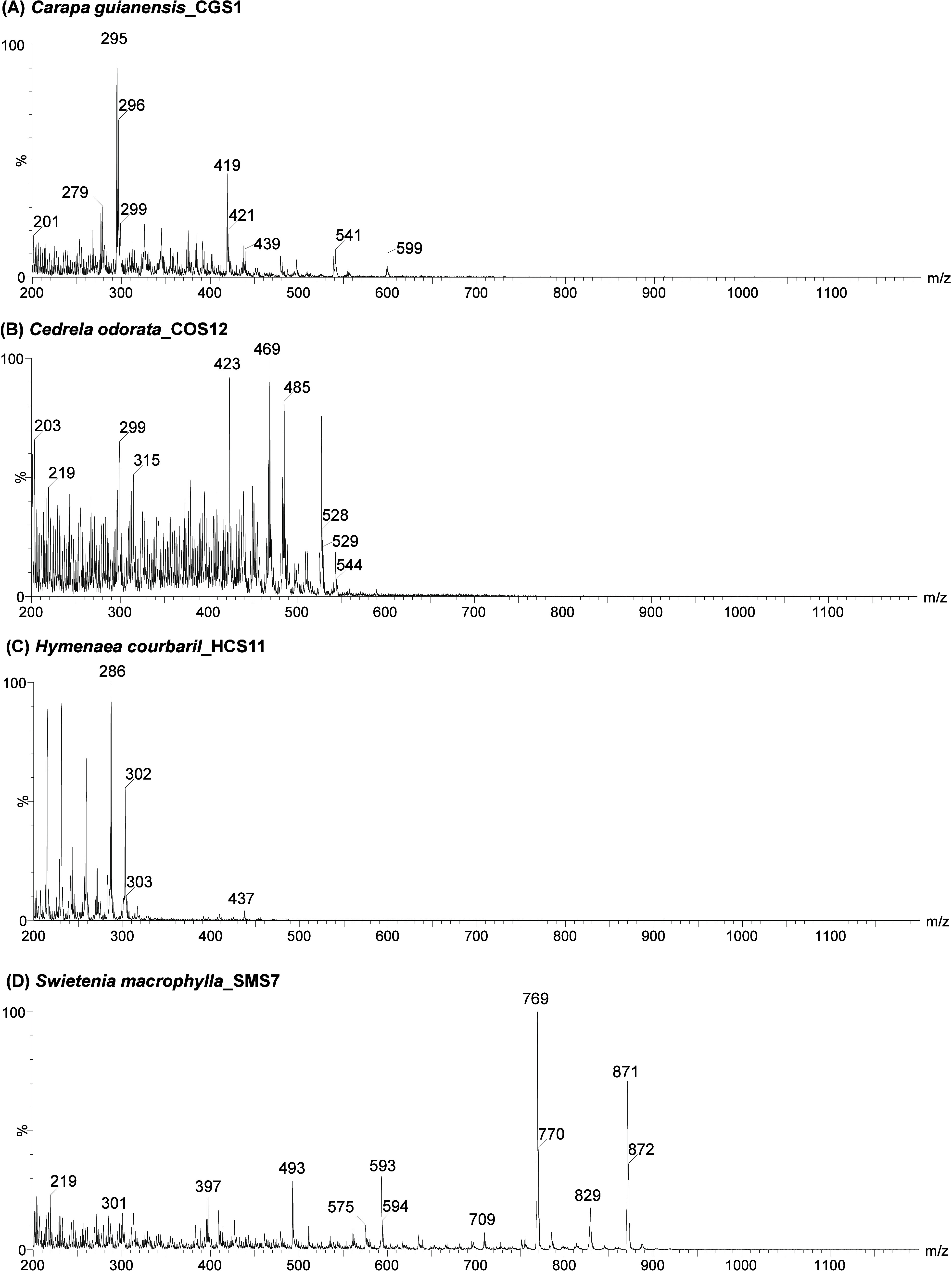
Representative full-scan mass spectra obtained in the low-resolution RADIAN ASAP mass spectrometer for samples of (A) *Carapa guianensis* (andiroba), (B) *Cedrela odorata* (cedar), (C) *Hymenaea courbaril* (jatoba), and (D) *Swietenia macrophylla* (mahogany).

**Table 2:** Most Abundant Ions in the Analyzed Wood Species

analyzed species	*m*/*z* of main most intense ions				
*Carapa guianensis* (andiroba)	279	295	419	541	599
*Cedrela odorata* (cedar)	423	467	469	485	527
*Hymenaea courbaril* (jatoba)	215	231	258	286	302
*Swietenia macrophylla* (mahogany)	493	593	769	829	871

During the analytical procedures, blank samples consisting solely of methanol:water (3:1) were analyzed to assess potential contamination within the analytical system. These blanks served to monitor the integrity of the instrumentation and to ensure that no external interference affected the spectral data obtained from the actual samples. Additionally, pool samples, composed of a representative mixture of extracts from all studied specimens, were analyzed to evaluate their spectral stability over the course of the analytical run. Also, the results showed that the signal intensities of the blanks were approximately 2 orders of magnitude lower than those of the real samples, confirming the absence of significant contamination. Furthermore, the spectra of the pool samples remained consistent across multiple runs, demonstrating the analytical system’s stability and repeatability.

### Xylem Anatomy

In the context of enforcement operations within the timber production chain, species identification is a critical factor in detecting fraud and illegal practices. It represents one of the key elements in ensuring the genuine sustainability of timber production. However, once the wood has been harvested, it is no longer accompanied by botanical structures, such as flowers, fruits, and leaves, which contain the primary taxonomic features used for the scientific classification of plant-derived materials.

Xylem (wood) anatomy thus becomes one of the few available alternatives for the taxonomic identification of timber, and it is the most widely used method worldwide for this purpose. However, due to the limited number of macroscopically visible features, its application requires substantial expertise and specialized knowledge from enforcement and regulatory personnel.^13,68^

Some groups of species exhibit striking similarities, either due to close phylogenetic relationships or evolutionary convergence, which result in comparable adaptations to the ecosystems they inhabit.^69^ This is particularly true for the species analyzed in this study, as they display similar organoleptic (color, smell, and texture) and anatomical characteristics (Figure 5). For anatomically similar wood species, reliable identification frequently depends on the expertise of a well-trained wood anatomist as misidentification can easily occur without specialized knowledge. Figure 5 shows the anatomical characteristics of representative samples of each species, emphasizing the striking resemblance in the organizational patterns of the three main xylem cell types: rays, vessels, and parenchyma. All four species exhibit growth rings delineated by marginal bands of axial parenchyma along with similarities in vessel size and vessel occlusion. They also share other traits such as comparable vessel frequency and distribution. Additionally, these species exhibit general similarities in density, hardness, and coloration. As a result, distinguishing among them is a complex task, especially when comparing andiroba (Figure 5A) and mahogany (Figure 5D), which possess nearly indistinguishable anatomical features—a challenge even for experienced wood anatomists. Consequently, macroscopic anatomical analyses conducted in the field may lead to erroneous species identification, even when they are performed by highly trained professionals.

**Figure 5:**
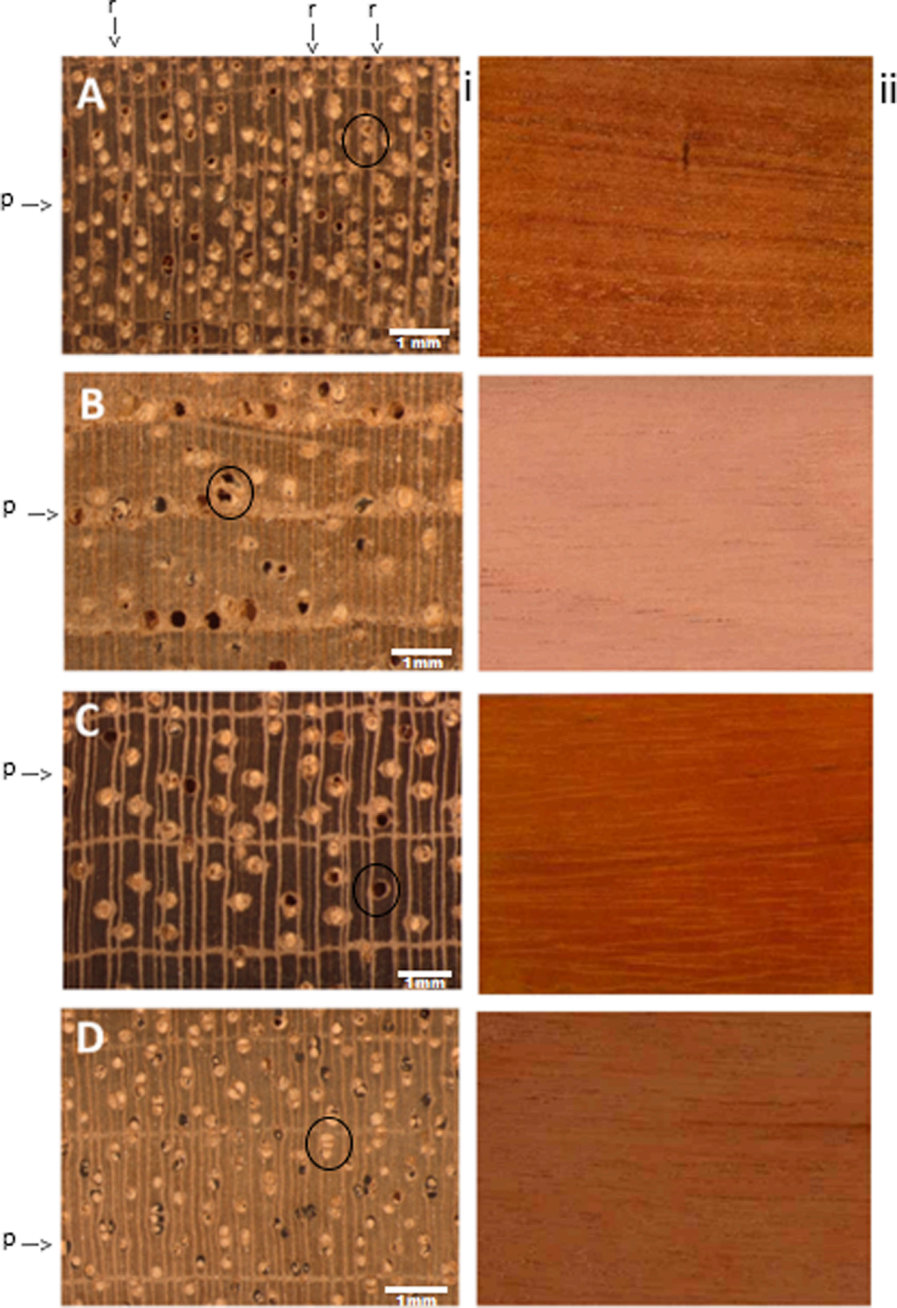
Macroscopic wood features of the four studied species. For each species (A–D), the left image displays the transverse surface imaged under a stereomicroscope at 10× magnification to reveal anatomical structures, while the right image shows the longitudinal surface without magnification (macroscopic view) to illustrate general texture and grain patterns: (A) *Carapa guianensis* (andiroba), (B) *Cedrela odorata* (cedar), (C) *Hymenaea courbaril* (jatoba), and (D) *Swietenia macrophylla* (mahogany). Abbreviations: r = rays; p = parenchyma; O = vessels.

These anatomical similarities create loopholes in the monitoring and control of timber supply chains, where restricted species such as mahogany are deliberately harvested and transported under the name “carapa”, in an attempt to circumvent regulatory oversight.^70^ The enforcement of regulations related to the harvesting, transportation, and commercialization of these species is an extremely challenging task influenced by several factors. Among them are the vast biodiversity of exploited timber species, the growing global demand for tropical hardwood, and, most critically, the shortage of trained personnel in wood anatomy within regulatory agencies.^71,72^

In this context, automated analytical tools capable of assisting species identification may complement traditional anatomical expertise in enforcement activities. Such technologies would enable regulatory personnel, without specialized training in xylem anatomy, to make more accurate and informed decisions regarding the monitoring and enforcement of the Brazilian tropical timber supply chain.

Moreover, considering that mahogany (*Swietenia macrophylla*) and cedar (*Cedrela odorata*) have been listed under Appendix II of CITES (Convention on International Trade in Endangered Species of Wild Fauna and Flora) since 2003 (IBAMA, 2022), which imposes restrictions on international trade and requires permits and strict origin control, the development of a characteristic spectral profile for these species may serve as a highly valuable tool for detecting illegal trade involving these regulated timbers.

### Multivariate Analysis

Statistical analysis of the results was performed by using LiveID software, which is integrated with the RADIAN ASAP system and designed to support automated analysis and identification in real time. The software employs chemometric models to compare mass spectrometry data against previously recorded spectra, enabling the classification and identification of unknown samples. LiveID facilitates the application of statistical methods to large and complex data sets, such as those generated by mass spectrometry, making it possible to distinguish between species or substances based on their chemical profiles.

Two of the most used statistical methods for sample classification based on the data generated by mass spectrometry are principal component analysis (PCA) and linear discriminant analysis (LDA). These methods are essential for transforming the complex and multivariate data obtained from spectrometry into useful information for the classification and identification of unknown samples.^73–75^ Principal component analysis (PCA) is an unsupervised multivariate analysis technique that transforms a large set of correlated variables into a smaller set of uncorrelated variables known as principal components. The main goal of PCA is to reduce data dimensionality while retaining the most relevant information, thereby facilitating visualization and interpretation of results.^77^ In contrast, linear discriminant analysis (LDA) is a supervised method used to find the linear combination of variables that best separates two or more predefined sample classes. Unlike PCA, LDA directly focuses on maximizing the differences between known groups, enhancing class separability.^76^

Various statistical methods have been employed in wood chemotyping, with results varying depending on the nature of the data and the specific objectives of the analysis. For instance, Brunswick et al. used the random forest machine learning algorithm to discriminate anatomical and chemical features of morphologically similar species within the *Dalbergia* genus. In parallel, they applied principal component analysis (PCA), which enabled the visualization of the separation of three *Dalbergia* species from other woods belonging to different genera, with 95% confidence.^77^ While PCA proved useful for visualizing variability and natural groupings in the data set, its limitation lies in being an unsupervised method, which may reduce its effectiveness in more complex classification tasks. In contrast, Lancaster and Espinoza employed both PCA and linear discriminant analysis (LDA) to identify 13 wood species, including *Dalbergia nigra*, a species classified as endangered. In this case, LDA yielded superior discriminative performance.^47^ As a supervised method, LDA is capable of maximizing the separation between predefined classes, making it particularly effective when differences between groups are subtle, as is often the case for species within the same genus. Complementarily, Yang et al. applied PCA and partial least squares discriminant analysis (PLS-DA) to differentiate between heartwood and sapwood in teak. Their results indicated that while PCA provided an initial indication of group separation, PLS-DA enabled clearer and more robust discrimination.^78^ This highlights the strength of PLS-DA as a powerful tool, especially when there is a linear relationship between the metabolites and sample classes.

The combined use of PCA and LDA is highly effective for the classification of the unknown samples. By integrating PCA and LDA, it is possible to build models based on known samples and subsequently classify unknown specimens with high accuracy. This statistical approach has proven to be particularly valuable in fields such as species authentication.^66,67,69^

Based on the sample analysis, a classification model was developed by combining principal component analysis (PCA) and linear discriminant analysis (LDA), resulting in a PCA-LDA model. The aim of this identification method is to differentiate classes based on instrumental measurements performed on samples, allowing for the real-time, online identification of new samples directly on the instrument. The model was built by using spectra obtained from triplicate analyses of each sample. Figure 6 illustrates the resulting PCA-LDA model, where a clear separation between sample groups can be observed, highlighting the technique’s strong potential for effective differentiation among these species.

**Figure 6:**
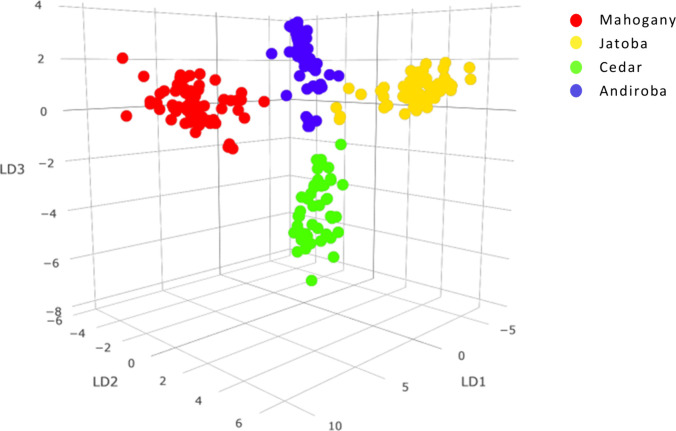
PCA-LDA model applied to samples of mahogany, jatoba, cedar, and andiroba.

Cross-validation of the PCA-LDA classification model yielded an accuracy of approximately 95%, with four misclassifications and eight samples identified as outliers (Table 3). A noteworthy case was that of jatoba, whose samples accounted for the majority of the outliers, making it the species with the highest number of outlier instances. This species exhibited more complex spectra, characterized by a high number of ions but lacking highly intense and distinctive marker ions, as observed in the other species. The absence of strong and specific signals made it more challenging for the model to accurately differentiate this species, leading to some misclassifications and causing the samples to deviate from their expected groupings. Note also that the sample size for *Hymenaea* (jatoba) specimens was reduced owing to the limited availability of suitable material in xylarium collections.

**Table 3:** Confusion Matrix and Results Summary for the PCA-LDA Model

analyzed species	andiroba	cedar	jatoba	mahogany	outlier	total
andiroba	61	1	3	0	2	67
cedar	0	45	0	0	0	45
jatoba	0	0	61	0	3	64
mahogany	0	0	0	69	3	72

results summary	spectra	passes	failures	outliers	correctness score
total	248	236	4	8	95.16%

Nevertheless, the overall performance of the model proved to be exceptional, particularly in the unequivocal classification of mahogany and cedar, which are the species protected by law.

To evaluate the model’s classification performance under simulated unknown-sample conditions, three samples from each species that had previously been excluded were reintroduced into the model for classification purposes. All samples were correctly identified with 100% confidence, as exemplified by the andiroba sample shown in Figure S7 of the Supporting Information.

This study clearly demonstrates the potential of mass spectrometry combined with multivariate analysis for reliable differentiation of wood species. Building on these results, future work will focus on expanding the spectral database to include a broader range of species with a particular emphasis on those that are endangered or vulnerable. The method shows strong promise as a valuable analytical tool for wood authentication, offering a low-cost, analytically robust approach. Its practical applicability suggests potential relevance for trade monitoring and enforcement activities related to illegal logging.

## Conclusions

The results demonstrated that the analysis of methanol/water (3:1) extracts is analytically viable using the proposed technique. However, the direct analysis of solid wood resulted in increased contamination of the mass spectrometer, favoring the use of solvent extracts as a more practical and instrument-friendly approach. Distinct chemical fingerprints were observed in the mass spectra of andiroba, mahogany, jatoba, and cedar, each exhibiting characteristic ion distributions in specific *m*/*z* regions. Chemometric modeling was performed using LiveID software, combining PCA and LDA to construct a robust classification model. The model, trained with triplicate spectra and validated, achieved over 95% accuracy. In the recognition test, all previously excluded samples were correctly classified with 100% confidence, confirming the method’s robustness and reliability for wood species chemotyping.

These results highlight the analytical potential of the RADIAN ASAP instrument, a compact, transportable single quadrupole mass spectrometer combined with multivariate analysis, to enable rapid and accurate chemical differentiation of wood species. The approach offers significant advancement over traditional anatomical methods, particularly when morphological features are obscured due to processing or degradation. As a direct, minimal sample preparation technique that delivers high-confidence classification, it represents an innovative contribution to wood chemotyping by using mass spectrometry.

Although the instrument is not portable in the conventional sense, this configuration provides operational flexibility and may allow installation in nontraditional laboratory environments and mobile units. Its minimal operational training requirements and infrastructure independence suggest potential utility for enforcement and regulatory agencies in supporting timber inspection activities. Future work will focus on expanding the spectral database to include additional species, particularly those that are threatened or frequently misidentified, with the aim of further evaluating and strengthening the method’s applicability to timber identification and regulatory contexts.

## Supplementary Material



## Notes

(Table S1) Set of samples analyzed using the RADIAN ASAP mass spectrometer, (Figure S1) full-scan mass spectra of andiroba (*Carapa guianensis*) samples, (Figure S2) full-scan mass spectra of cedar (*Cedrela odorata*) samples, (Figure S3) full-scan mass spectra of jatoba (*Hymenaea courbaril*) samples, (Figure S4) full-scan mass spectra of mahogany (S*wietenia macrophylla*) samples, (Figure S5) full-scan mass spectra of representative blank samples, (Figure S6) full-scan mass spectra of representative pool samples and Figure S7, example of LiveID software recognizing the Andiroba sample in real time (PDF)
