# Supplementary material for: Real-Time Wood Chemotyping Using a Low-Cost and Compact Mass Spectrometer
Source: ACS Omega. 2026 Mar 5;11(10):16687–99. doi: 10.1021/acsomega.5c13161 (PMC13000584; doi:10.1021/acsomega.5c13161)

SUPPORT INFORMATION

REAL-TIME WOOD CHEMOTYPING USING A LOW-COST AND COMPACT MASS SPECTROMETER

Authors: Thays V. C. Monteiro[1], Mariana Fioramonte[2], Renan Pirolla[2], Alexandre Bahia Gontijo[3,4], Cristiano Nascimento[5], Niro Higuchi[5], Mário Augusto Gonçalves Jardim[6], Maíra Fasciotti [1*]

1 Inmetro – National Institute of Metrology, Quality and Technology – Duque de Caxias, Brazil.
2 Waters – Waters Technologies do Brasil – São Paulo, Brazil.
3 LPF/SFB – Laboratório de Produtos Florestais/Serviço Florestal Brasileiro – Brasília, Brazil.
4 University of Brasília, Institute of Biological Sciences, Graduate Program in Ecology, Distrito Federal, Brazil, 70910-900
5 LMF/INPA – Forest Management Laboratory/Instituto Nacional de Pesquisas da Amazônia – Amazonas, Brazil.
6 Museu Goeldi – Paraense Emílio Goeldi Museum/Coordination of Botany – Pará, Brazil.

Corresponding author: Dr. Maíra Fasciotti, mfasciotti@inmetro.gov.br

Table S1. Set of samples analyzed using the Radian ASAP mass spectrometer.

**Species**	**Popular name**	**Institution**	**Internal code**	**Part of the trunk**	**Collection location**
Carapa guianensis	Andiroba	LPF	CGS1	Heartwood/ sap-wood	Flona Tapajós - BR 163 - Satarém/PA
Carapa guianensis	Andiroba	LPF	CGS3	Heartwood	Flona Tapajós - BR 163 - Satarém/PA
Carapa guianensis	Andiroba	LPF	CGS4	Heartwood	Flona Tapajós - BR 163 - Satarém/PA
Carapa guianensis	Andiroba	LPF	CGS5	Heartwood	Flona Tapajós - BR 163 - Satarém/PA
Carapa guianensis	Andiroba	LPF	CGS6	Heartwood	Flona Tapajós - BR 163 - Satarém/PA
Carapa guianensis	Andiroba	LPF	CGS7	Heartwood	Flona Tapajós - BR 163 - Satarém/PA
Carapa guianensis	Andiroba	LPF	CGS8	Heartwood	Flona Tapajós - BR 163 - Satarém/PA
Carapa guianensis	Andiroba	INPA	CGS9	Heartwood	Presidente Figueiredo - Amazonas
Carapa guianensis	Andiroba	INPA	CGS10	Heartwood/ sap-wood	Itacoatiara - Amazonas
Carapa guianensis	Andiroba	INPA	CGS11	Heartwood/ sap-wood	Itacoatiara - Amazonas
Carapa guianensis	Andiroba	INPA	CGS12	Heartwood	Manaus - Amazonas
Carapa guianensis	Andiroba	INPA	CGS13	Heartwood	Manaus - Amazonas
Carapa guianensis	Andiroba	Goeldi	CGS14	No information	Pará - Rio Tocantins
Carapa guianensis	Andiroba	Goeldi	CGS15	No information	Pará - BR-422
Carapa guianensis	Andiroba	Goeldi	CGS16	No information	Venezuela
Carapa guianensis	Andiroba	LPF	CGS17	Heartwood	No information
Carapa guianensis	Andiroba	LPF	CGS18	Heartwood	No information
Carapa guianensis	Andiroba	LPF	CGS19	Heartwood	No information
Cedrela odorata	Cedar	LPF	COS1	Heartwood	Flona Jamari - Jamari/RO
Cedrela odorata	Cedar	UFRRJ	COS2	No information	Pará
Cedrela odorata	Cedar	LPF	COS3	No information	Flona Jamari - Jamari/RO
Cedrela odorata	Cedar	INPA	COS4	Heartwood/ sap-wood	Santárem- Pará
Cedrela odorata	Cedar	INPA	COS5	Heartwood/ sap-wood	Presidente Figueiredo - Amazonas
Cedrela odorata	Cedar	INPA	COS6	Heartwood/ sap-wood	Itacoatiara - Amazonas
Cedrela odorata	Cedar	INPA	COS7	Heartwood/ sap-wood	Itacoatiara - Amazonas
Cedrela odorata	Cedar	INPA	COS8	Heartwood/ sap-wood	Itacoatiara - Amazonas

Cedrela odorata	Cedar	INPA	COS9	Heartwood/ sap-wood	Itacoatiara - Amazonas
Cedrela odorata	Cedar	Goeldi	COS10	No information	Rondônia - BR-364
Cedrela odorata	Cedar	Goeldi	COS11	No information	Pará - Rio Iriri
Cedrela odorata	Cedar	Goeldi	COS12	No information	Pará - Campus Embrapa-Belém
Cedrela odorata	Cedar	LPF	COS13	Heartwood	No information
Cedrela odorata	Cedar	LPF	COS14	Heartwood	No information
Cedrela odorata	Cedar	LPF	COS15	Heartwood	No information
Hymenaea courbaril	Jatobá	LPF	HCS1	Heartwood/ sap-wood	Jari/PA
Hymenaea courbaril	Jatobá	UFRRJ	HCS2	No information	Pará
Hymenaea courbaril	Jatobá	INPA	HCS5	Heartwood	Itacoatiara - Amazonas
Hymenaea courbaril	Jatobá	INPA	HCS6	Heartwood	Itacoatiara - Amazonas
Hymenaea courbaril	Jatobá	INPA	HCS7	Heartwood	Itacoatiara - Amazonas
Hymenaea courbaril	Jatobá	Goeldi	HCS8	No information	Mato Grosso - BR-174
Hymenaea courbaril	Jatobá	Goeldi	HCS9	No information	Suriname - Rio Saramacco
Hymenaea courbaril	Jatobá	Goeldi	HCS10	No information	Pará
Hymenaea courbaril	Jatobá	LPF	HCS11	Heartwood	No information
Hymenaea courbaril	Jatobá	LPF	HCS12	Heartwood	No information
Hymenaea courbaril	Jatobá	LPF	HCS13	Heartwood	No information
Swietenia macrophylla	Mahogany	LPF	SMS1	Heartwood	No information
Swietenia macrophylla	Mahogany	UFRRJ	SMS2	No information	Peru (Amazônia)
Swietenia macrophylla	Mahogany	No information	SMS3	No information	No information
Swietenia macrophylla	Mahogany	No information	SMS4	No information	No information
Swietenia macrophylla	Mahogany	No information	SMS5	No information	No information
Swietenia macrophylla	Mahogany	INPA	SMS6	Heartwood	Santarém - Pará
Swietenia macrophylla	Mahogany	INPA	SMS7	Heartwood	Presidente Figueiredo - Amazonas
Swietenia macrophylla	Mahogany	INPA	SMS8	Heartwood	Rondônia
Swietenia macrophylla	Mahogany	INPA	SMS9	Heartwood	Amazonas
Swietenia macrophylla	Mahogany	INPA	SMS10	Heartwood	Amazonas
Swietenia macrophylla	Mahogany	INPA	SMS11	Heartwood	Amazonas
Swietenia macrophylla	Mahogany	INPA	SMS12	Heartwood	Amazonas
Swietenia macrophylla	Mahogany	INPA	SMS13	Heartwood	Amazonas
Swietenia macrophylla	Mahogany	INPA	SMS14	Heartwood	Amazonas
Swietenia macrophylla	Mahogany	INPA	SMS15	Heartwood	Amazonas
Swietenia macrophylla	Mahogany	Goeldi	SMS16	No information	Pará

Figure S1. Full scan mass spectra of andiroba (*Carapa guianensis*) samples.

(A) CGS1

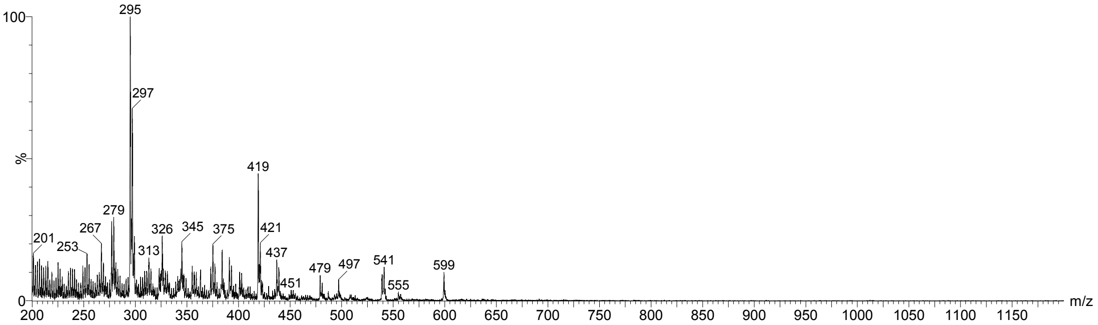

(B) CGS3

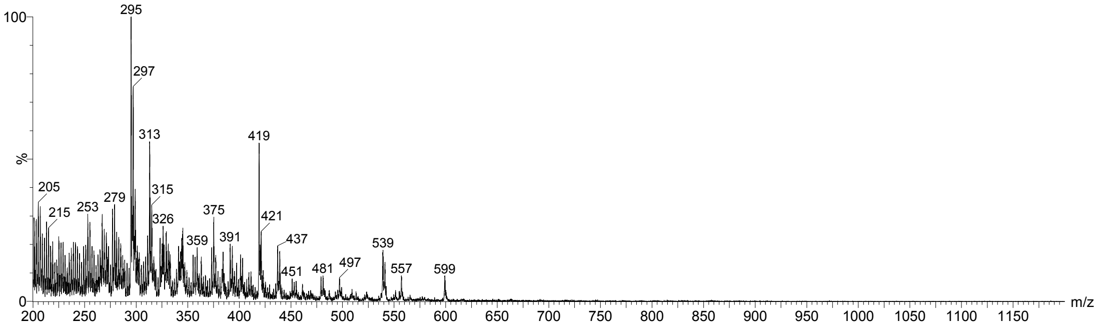

(C) CGS4

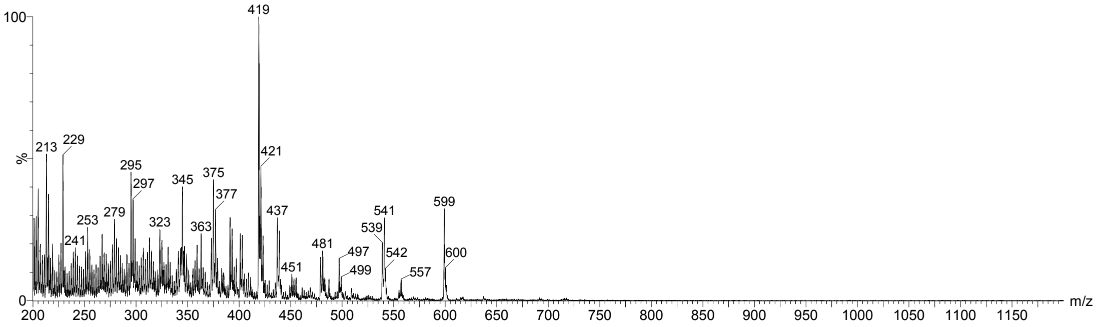

(D) CGS5

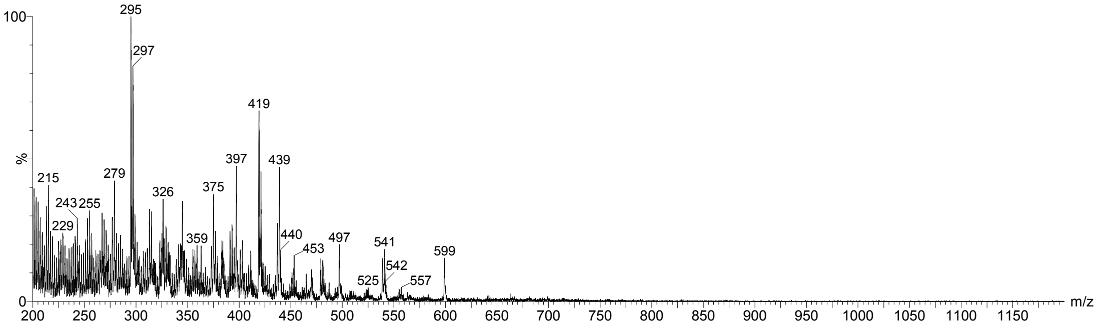

(E) CGS6

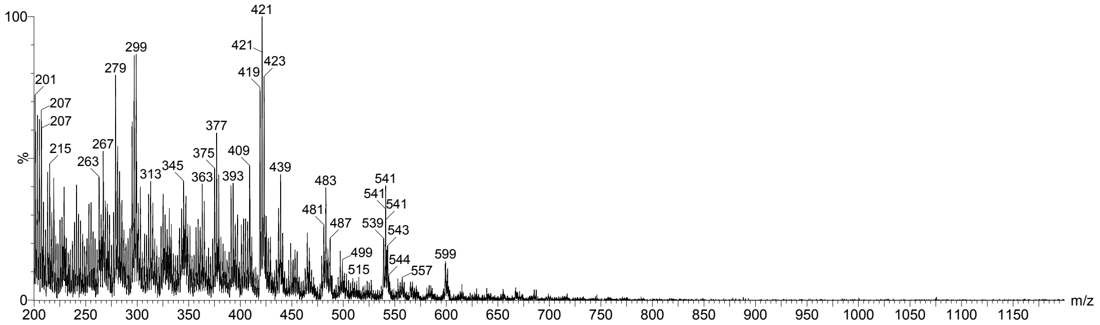

(F) CGS7

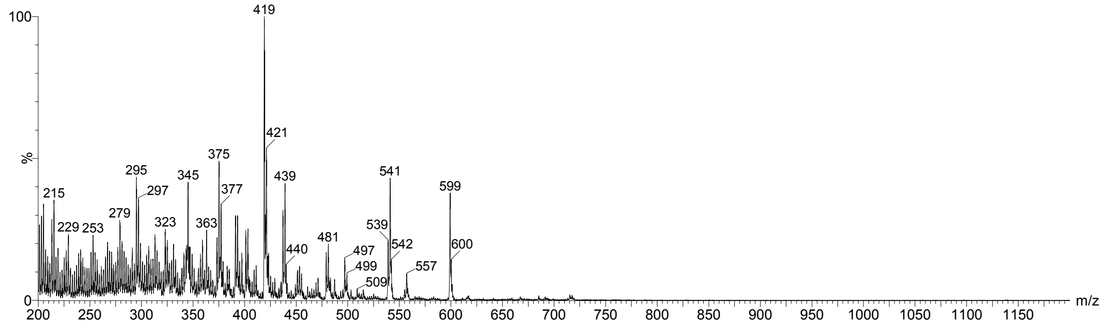

(G) CGS8

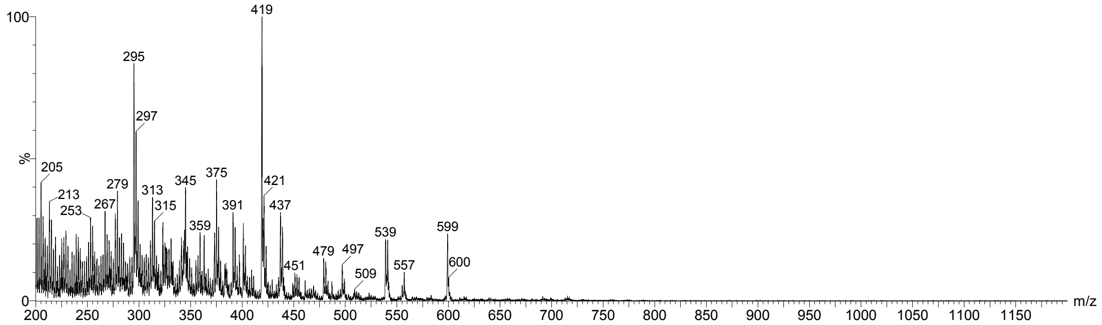

(H) CGS9

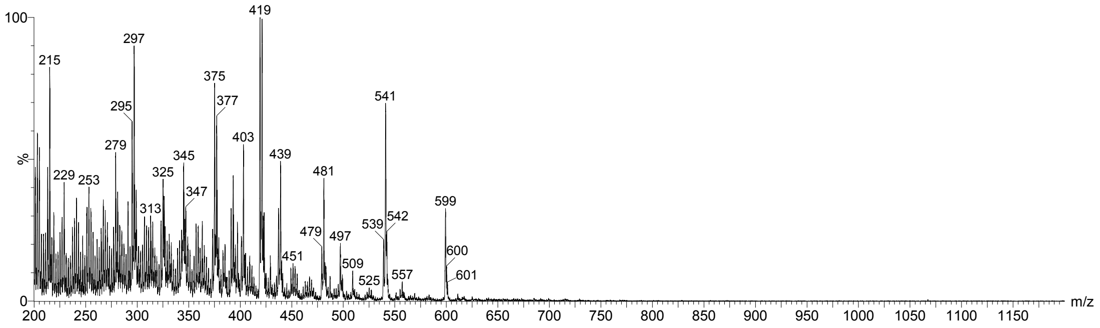

(I) CGS10

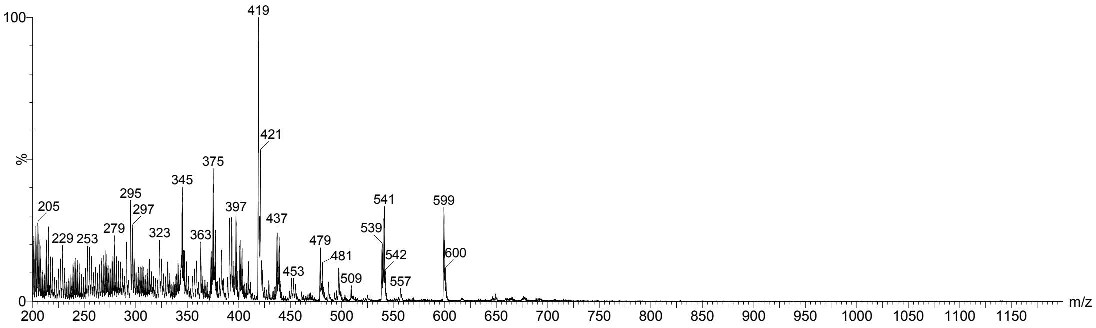

(J) CGS11

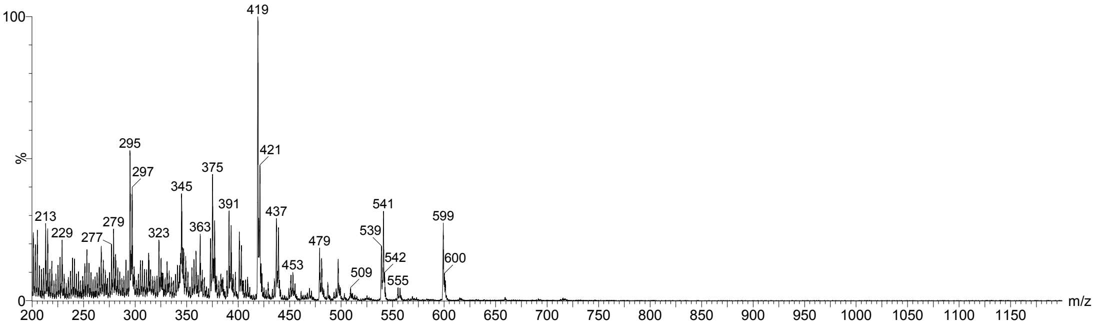

(K) CGS12

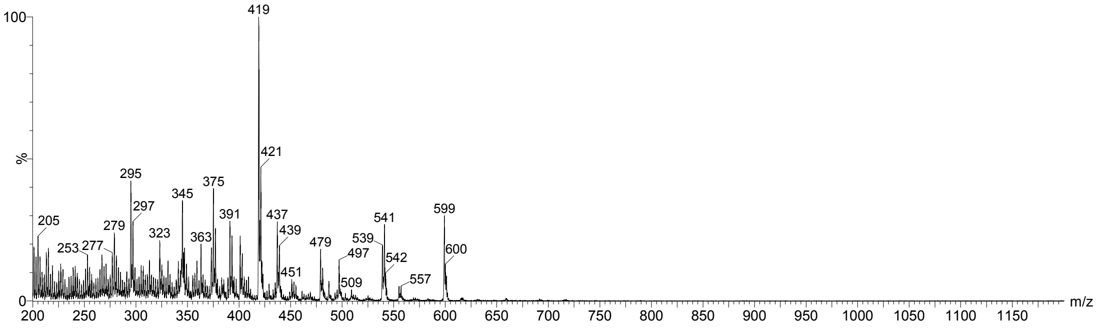

(L) CGS13

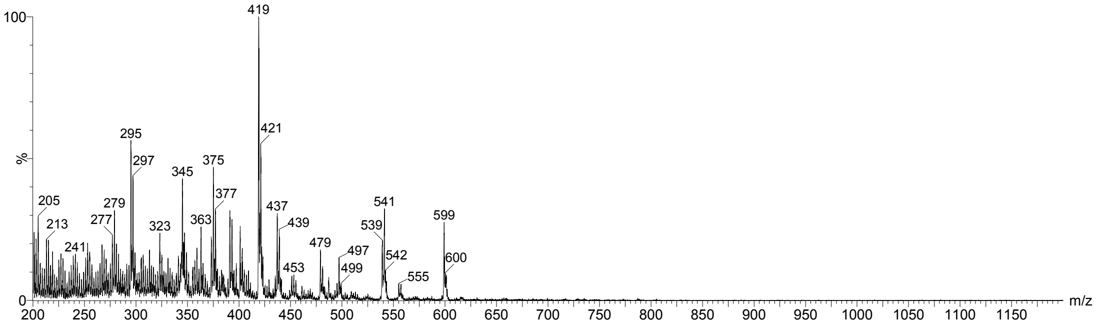

(M) CGS14

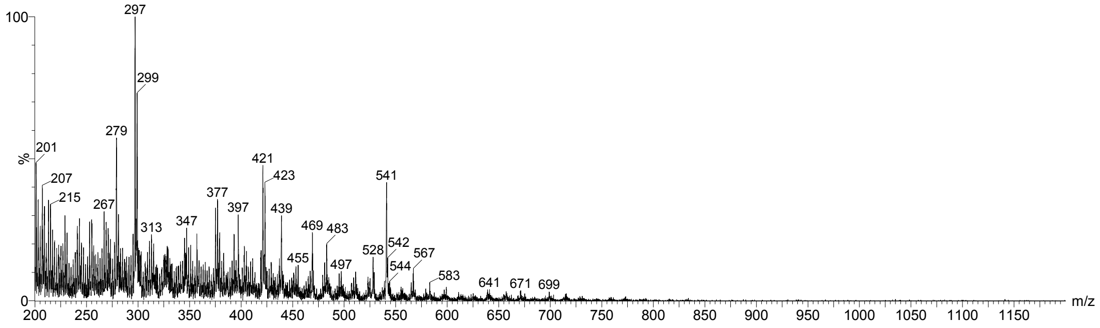

(N) CGS15

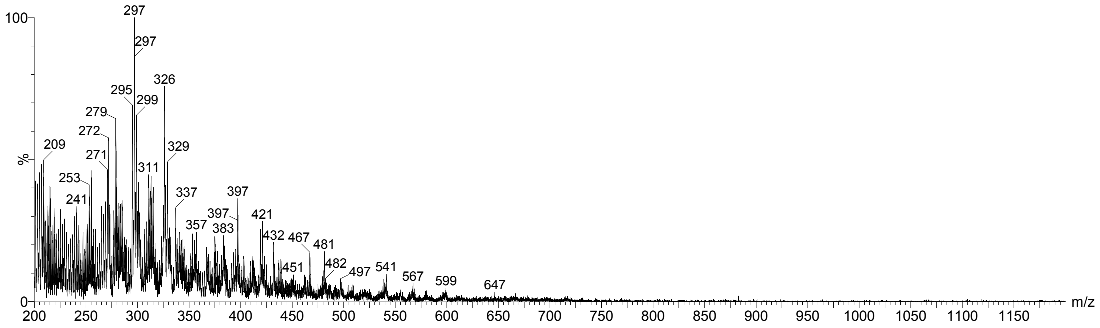

(O) CGS16

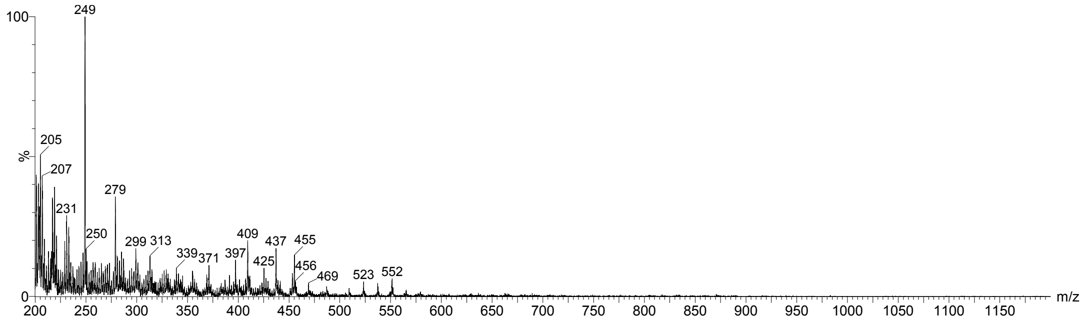

(P) CGS17

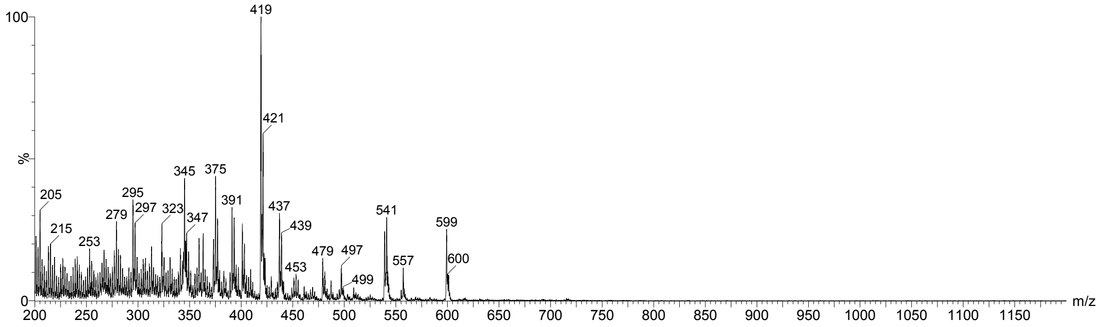

(Q) CGS18

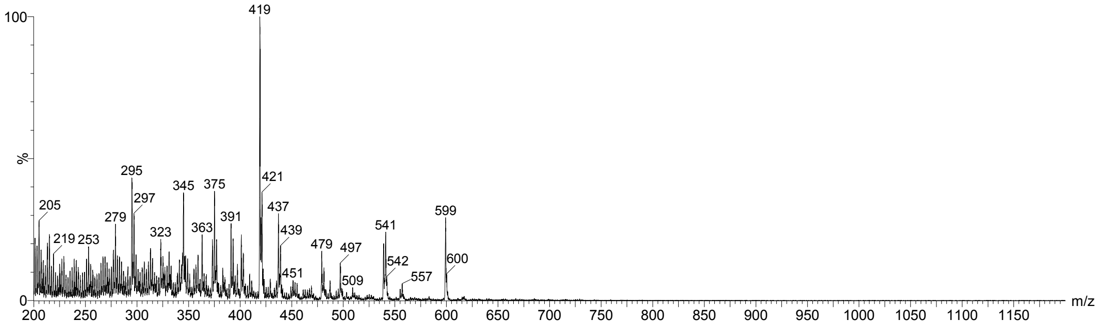

(R) CGS19

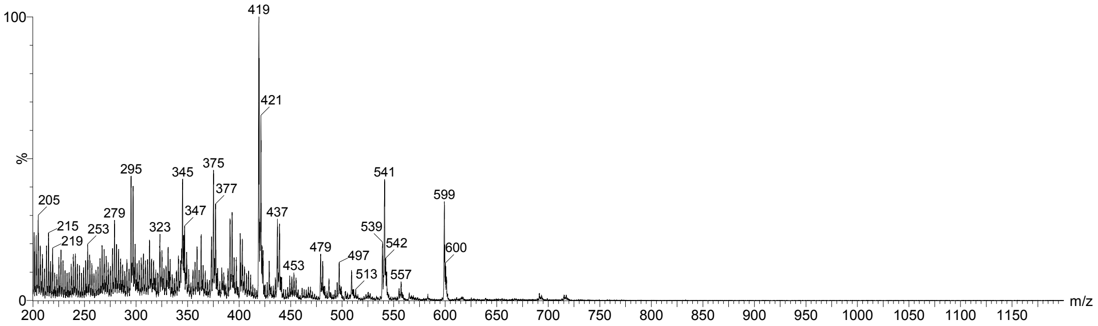

Figure S2. Full scan mass spectra of cedar (*Cedrela odorata*) samples.

(A) COS1

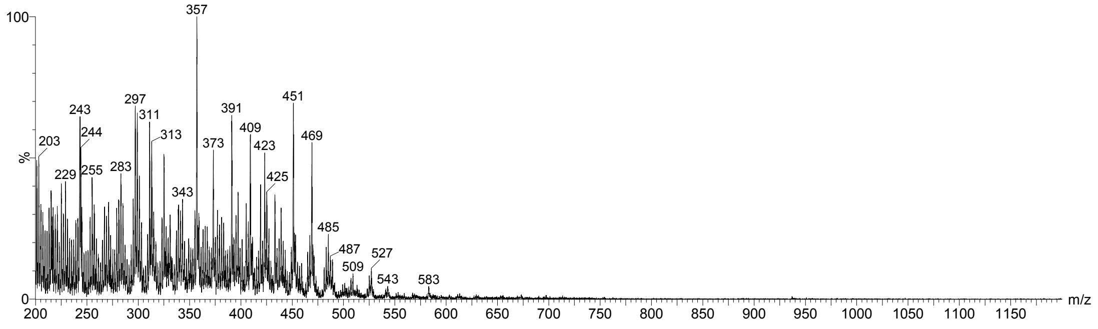

(B) COS2

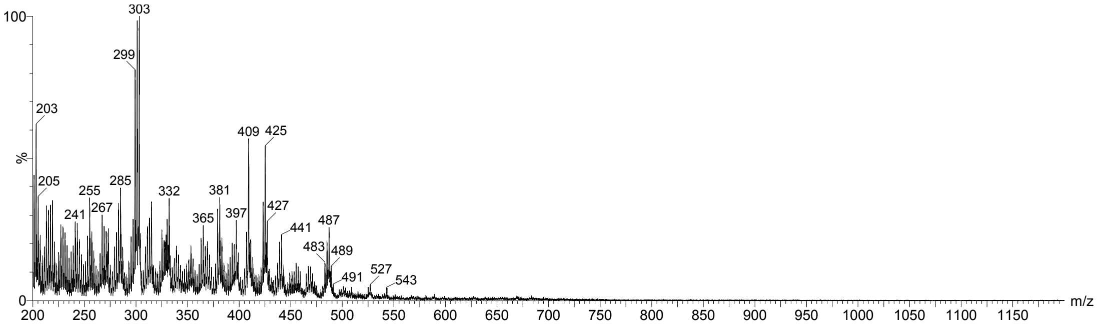

(C) COS3

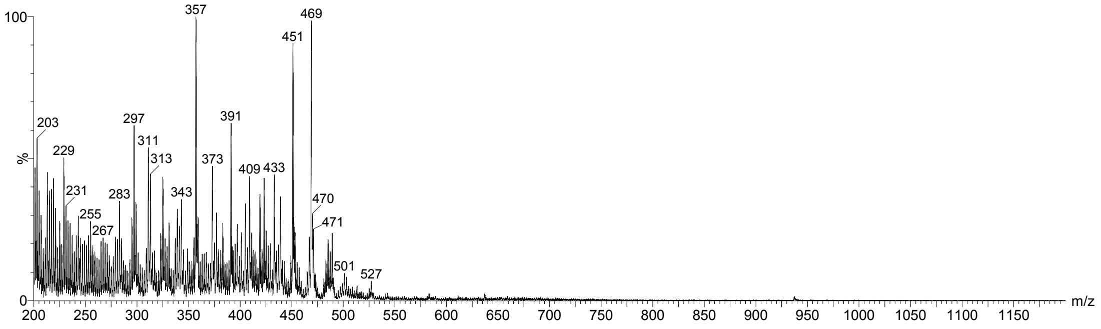

(D) COS4

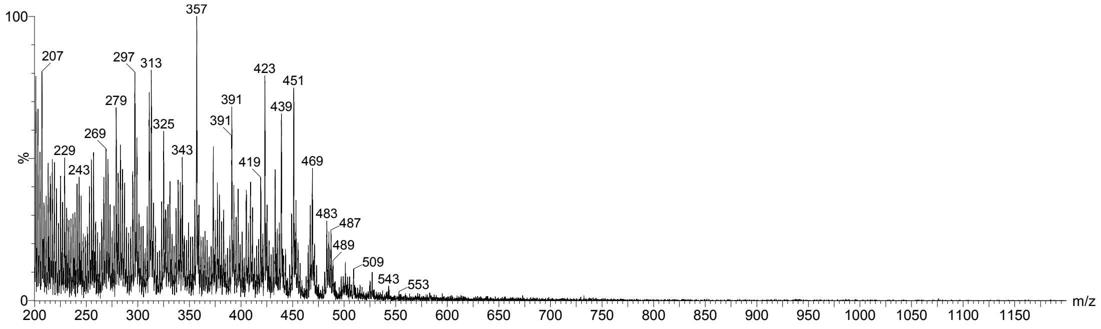

(E) COS5

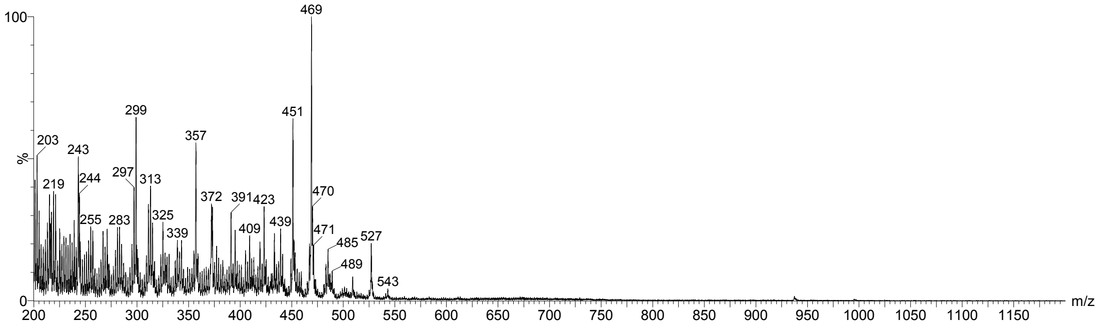

(F) COS6

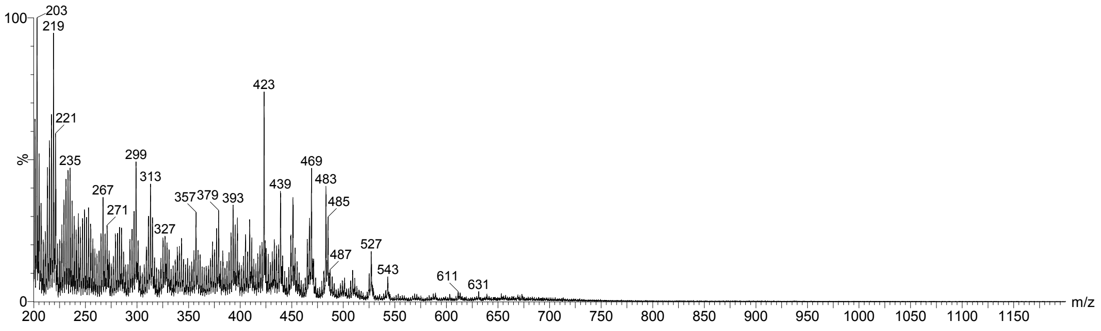

(G) COS7

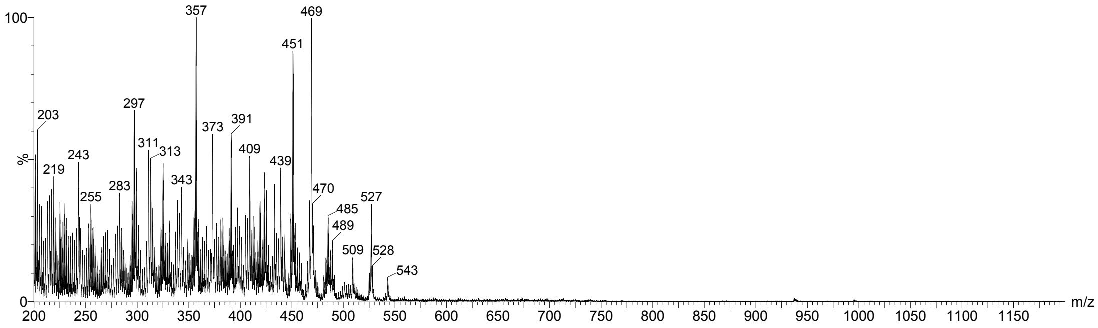

(H) COS8

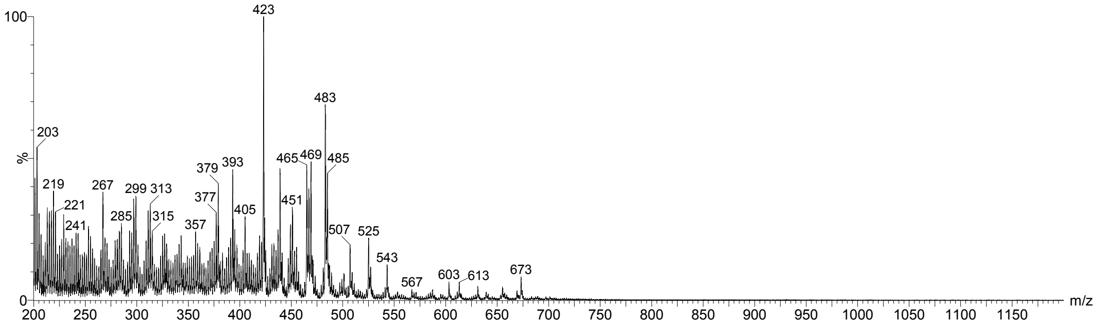

(I) COS9

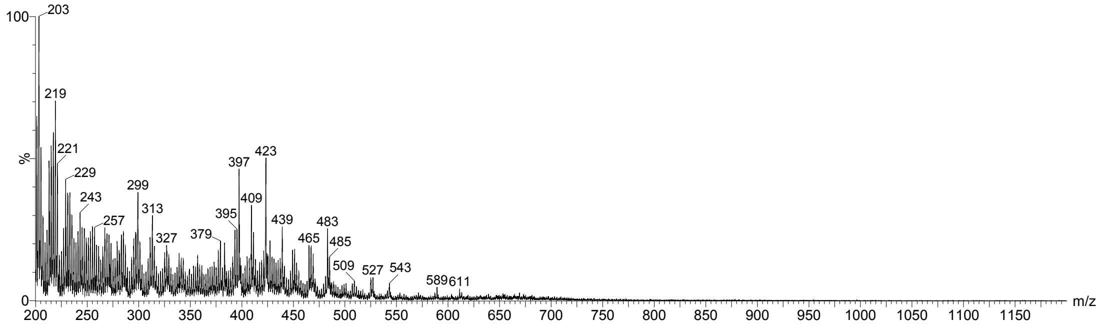

(J) COS10

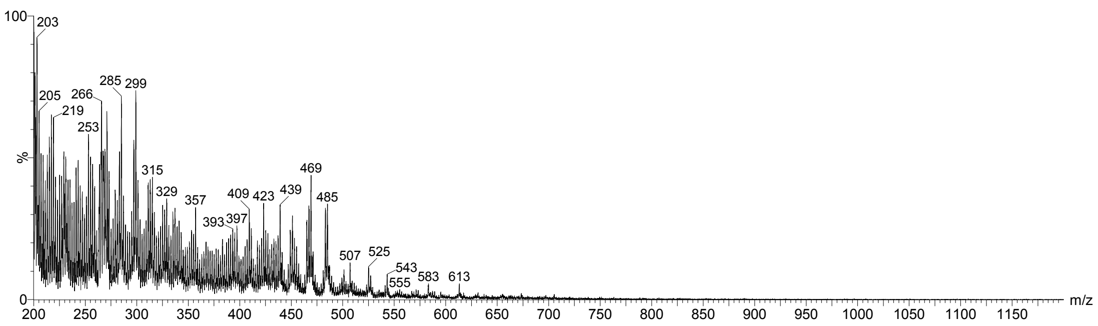

(K) COS11

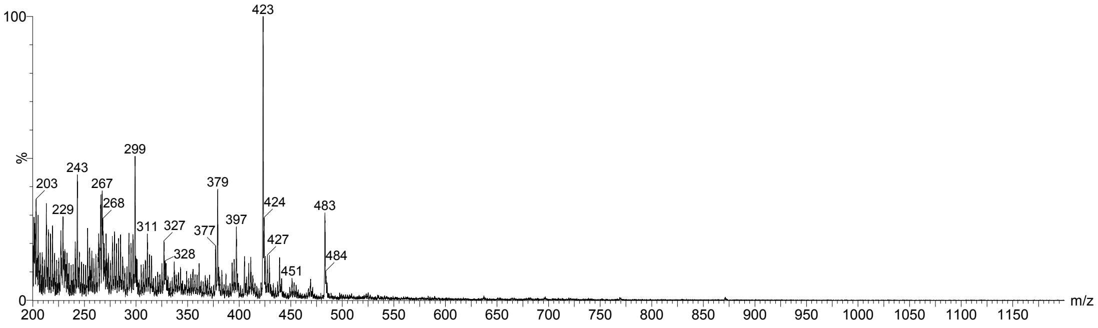

(L) COS12

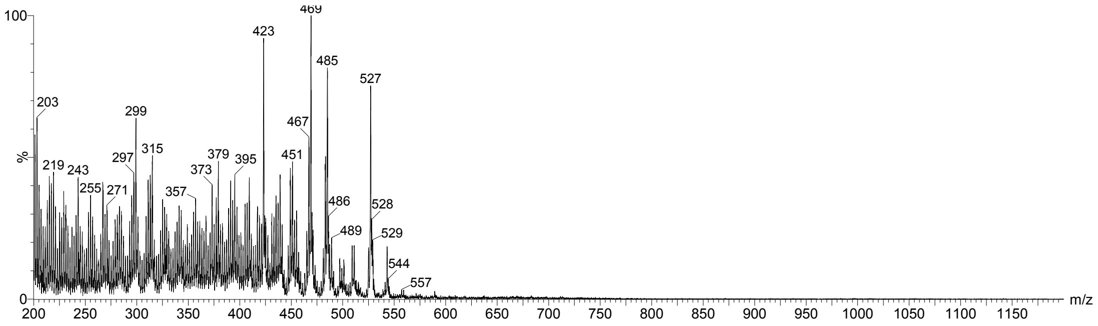

(M) COS13

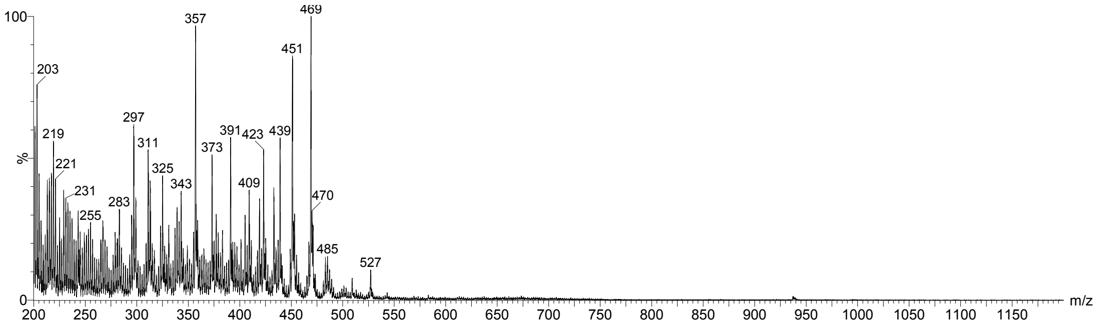

(N) COS14

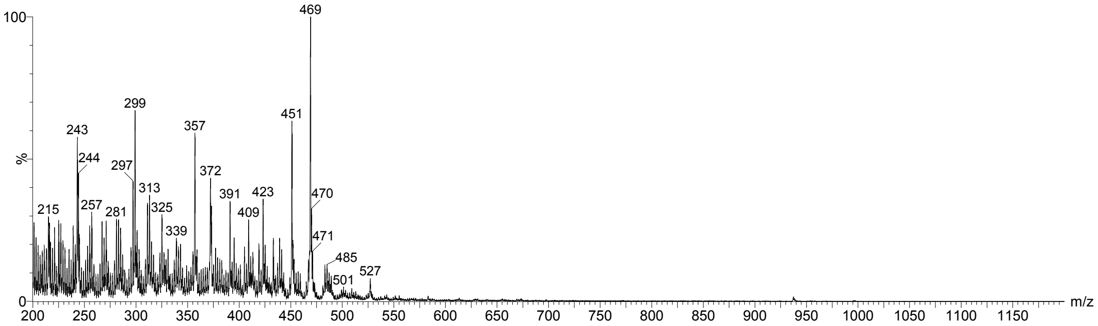

(O) COS15

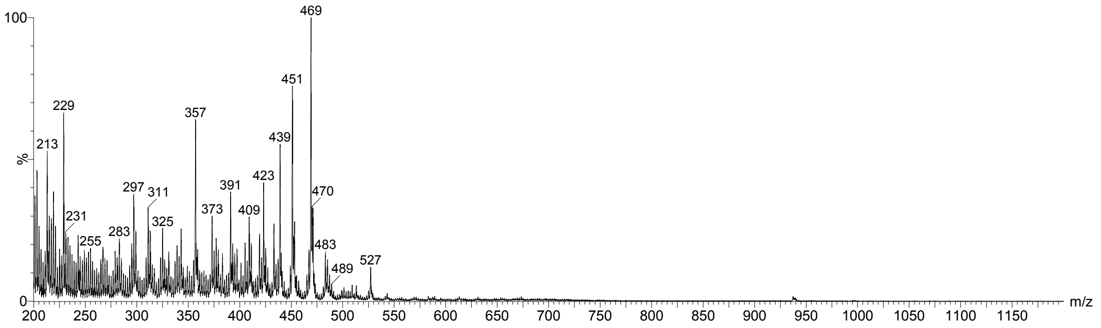

Figure S3. Full scan mass spectra of jatobá (*Hymenaea courbaril*) samples.

(A) HCS1

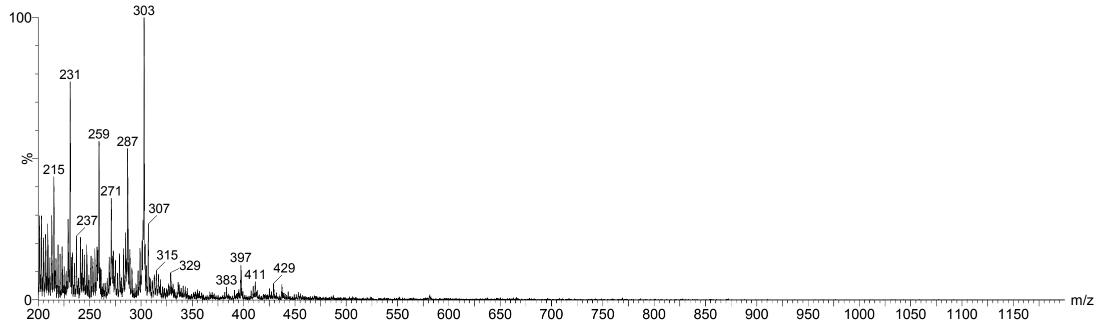

(B) HCS2

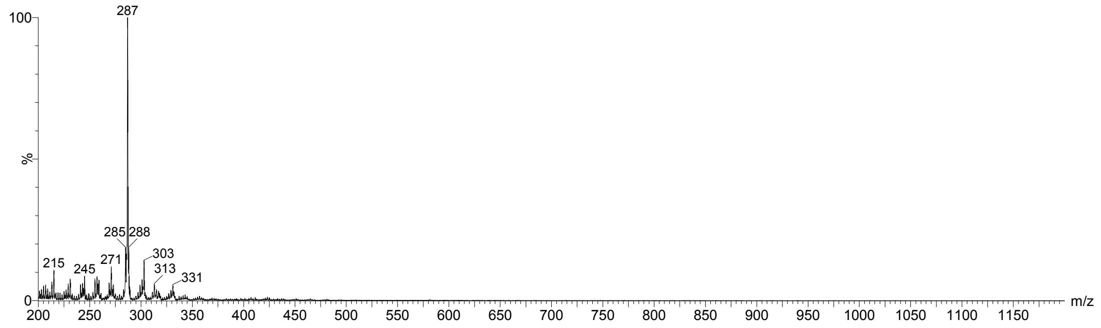

(C) HCS5

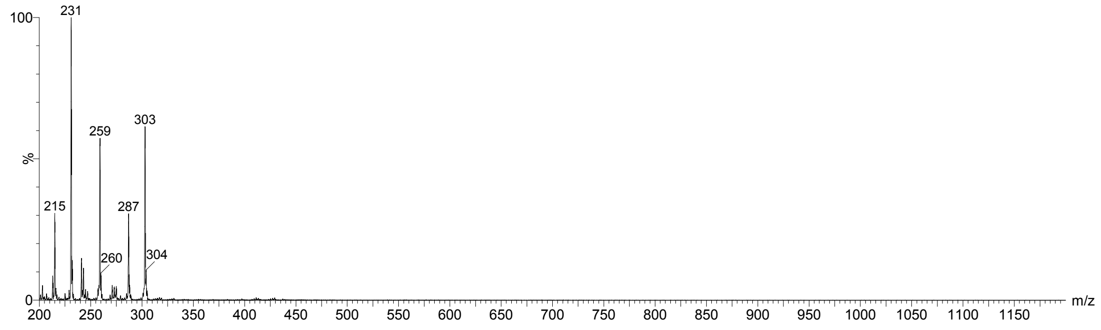

(D) HCS6

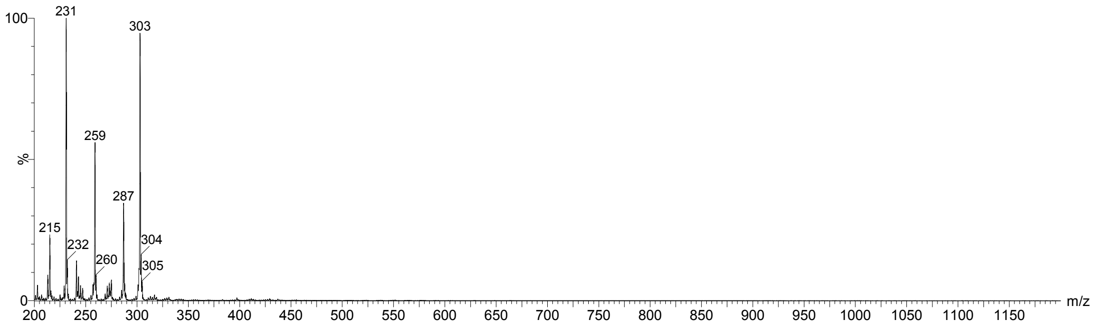

(E) HCS7

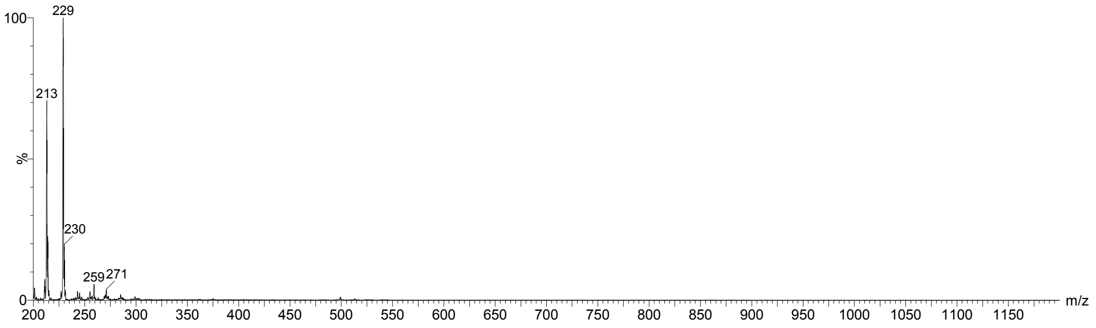

(F) HCS8

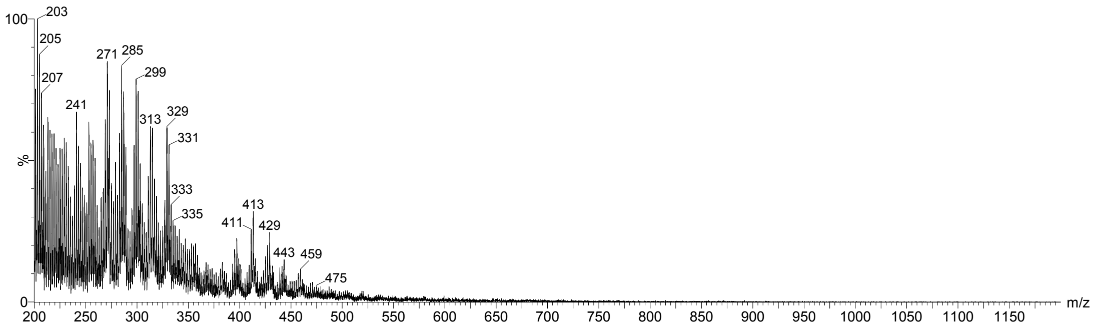

(G) HCS9

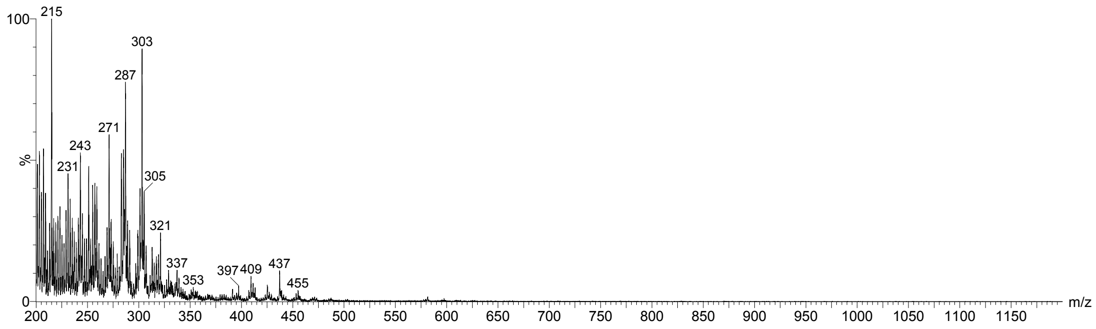

(H) HCS10

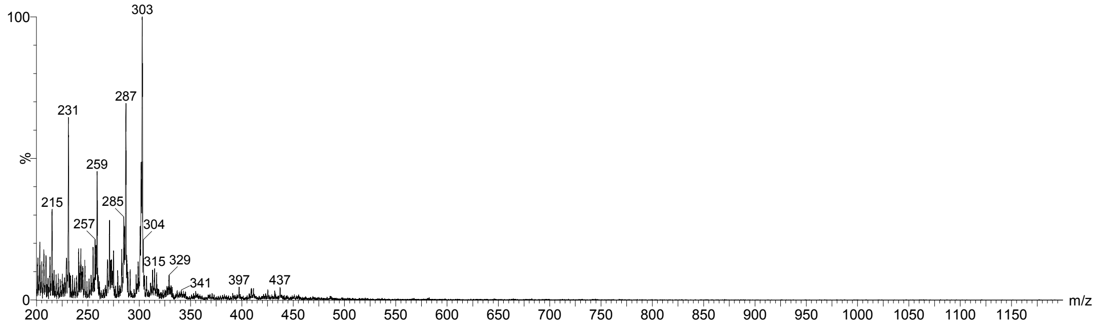

(I) HCS11

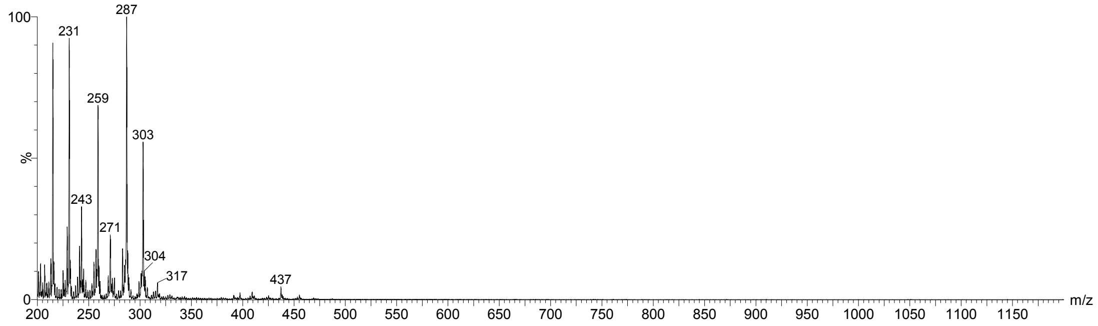

(J) HCS12

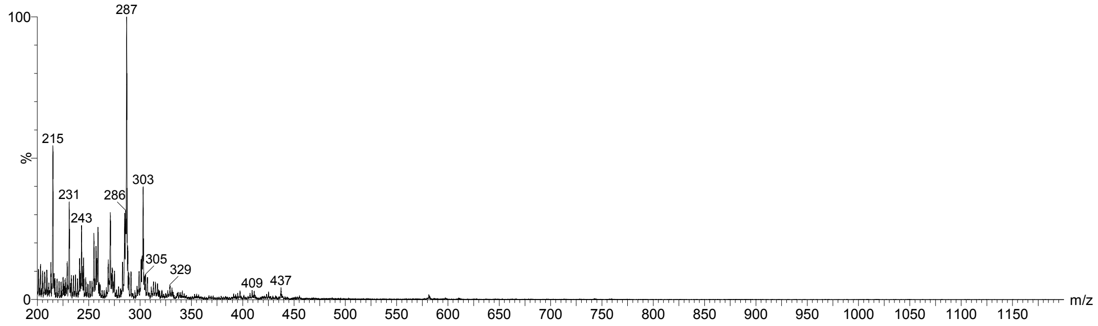

(K) HCS13

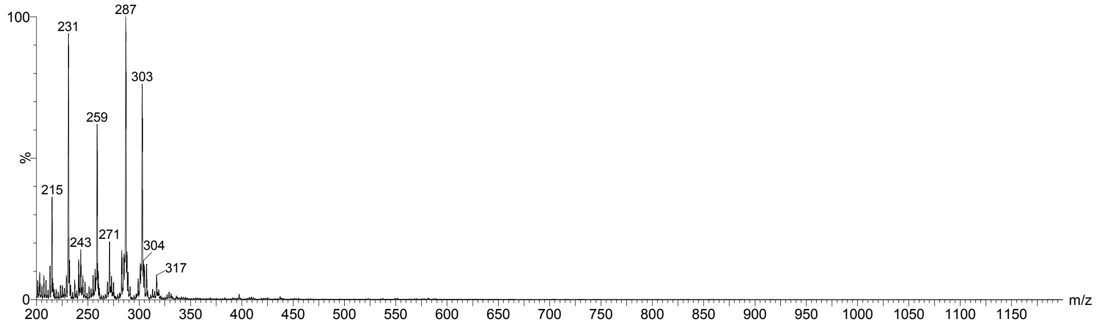

Figure S4. Full scan mass spectra of mahogany (S*wietenia macrophylla*) samples.

(A) SMS1

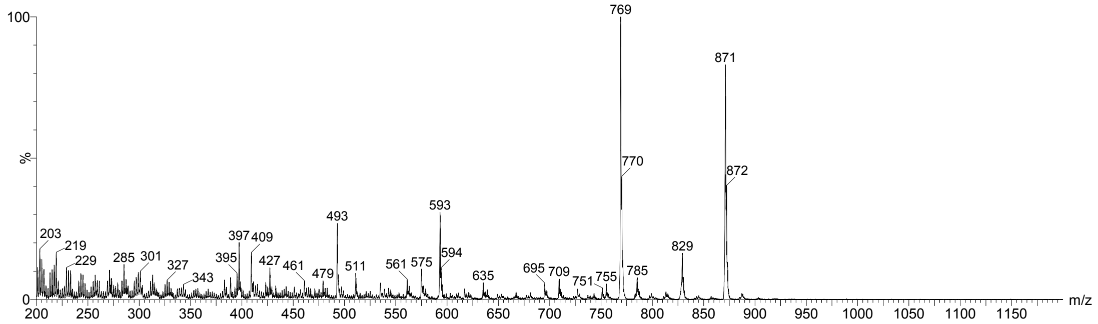

(B) SMS2

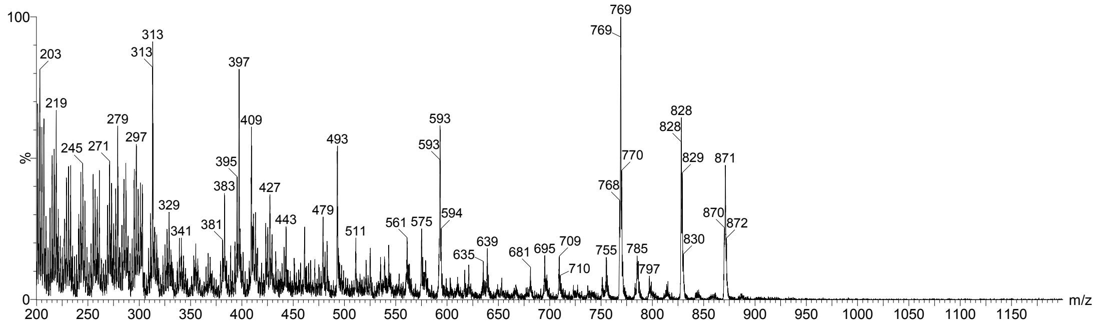

(C) SMS3

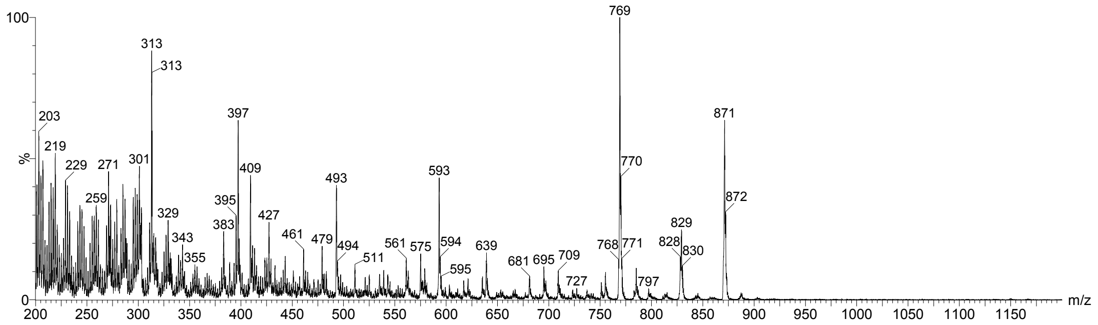

(D) SMS4

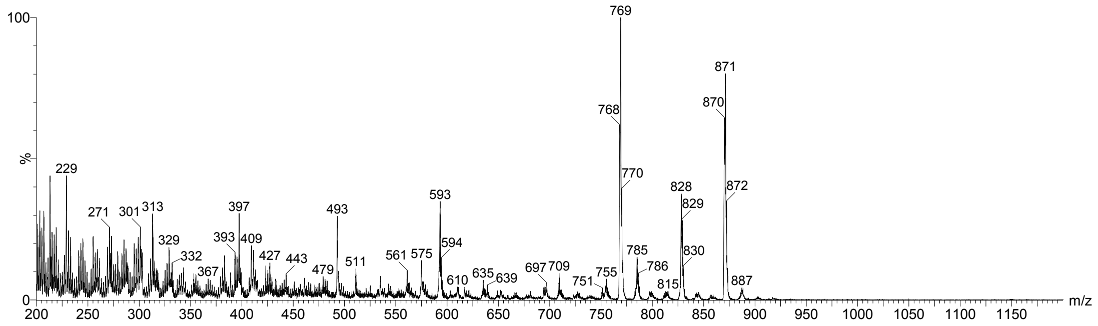

(E) SMS5

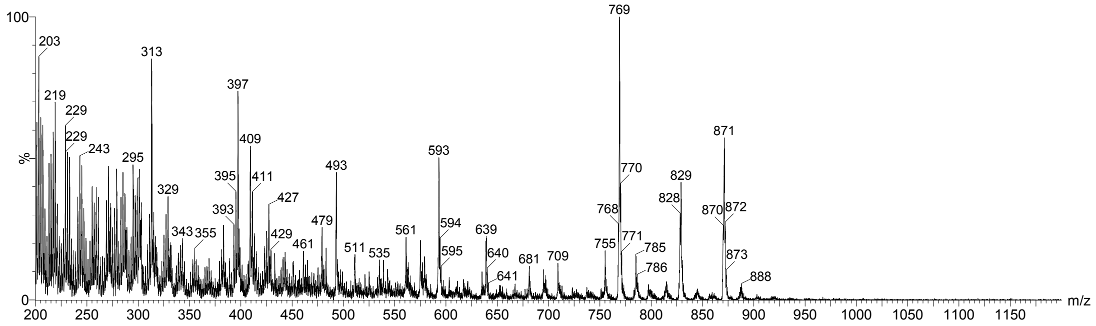

(F) SMS6

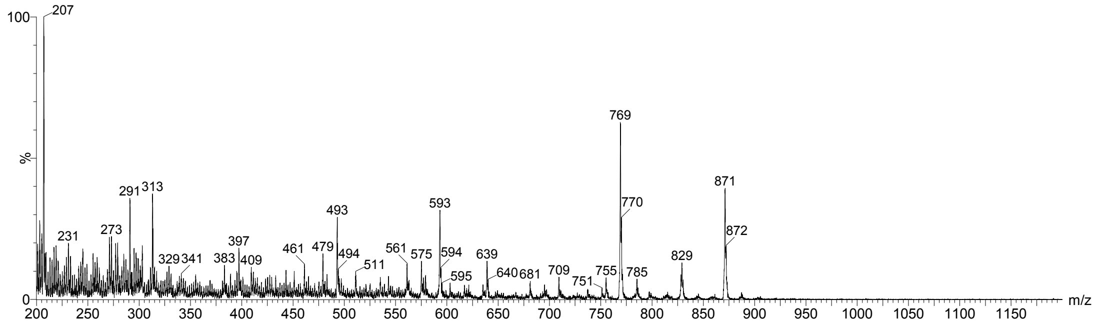

(G) SMS7

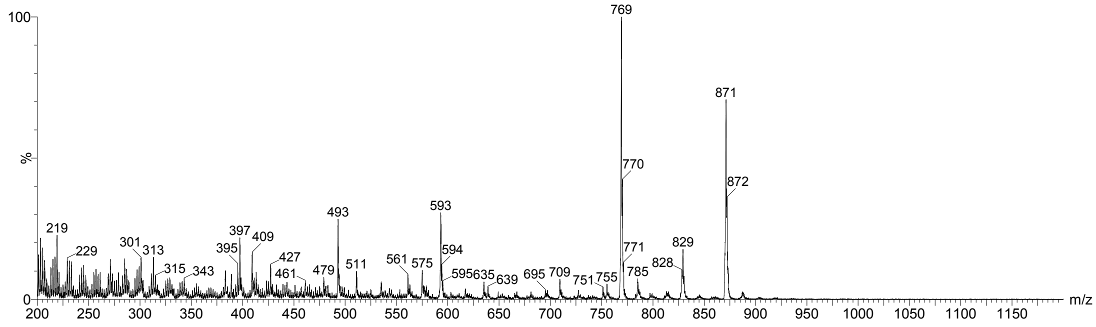

(H) SMS8

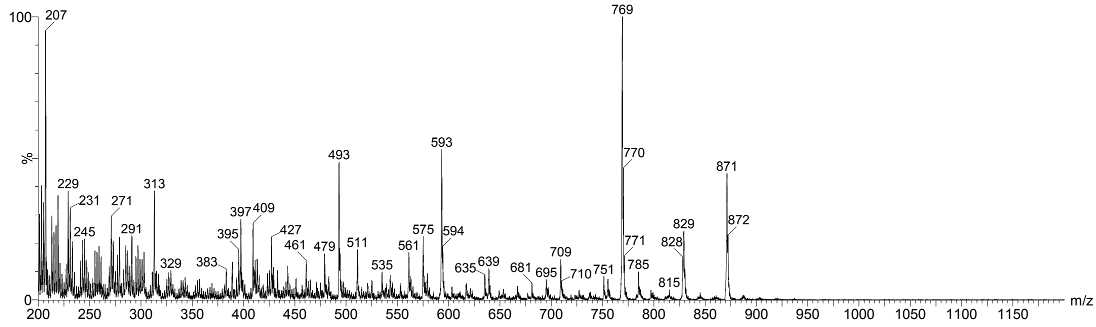

(I) SMS9

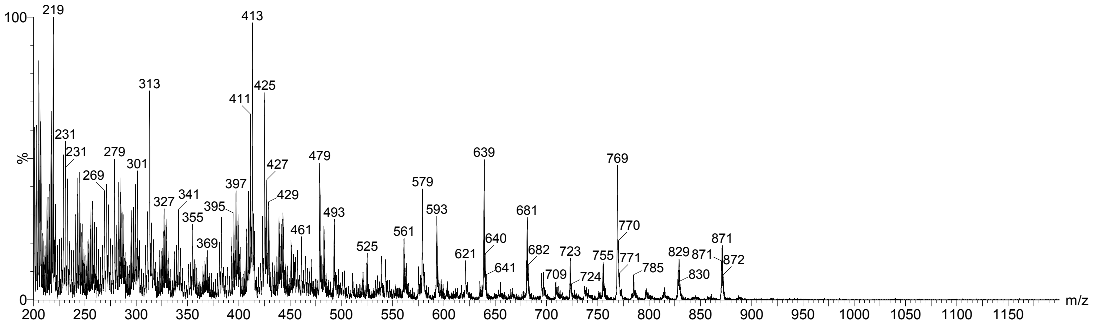

(J) SMS10

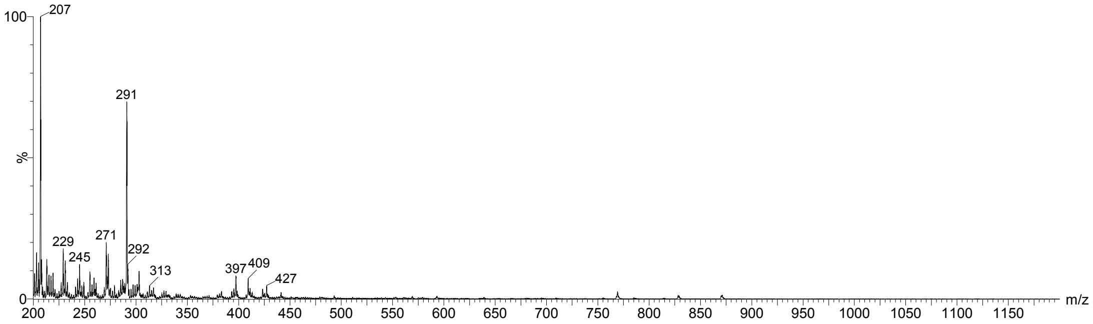

(K) SMS11

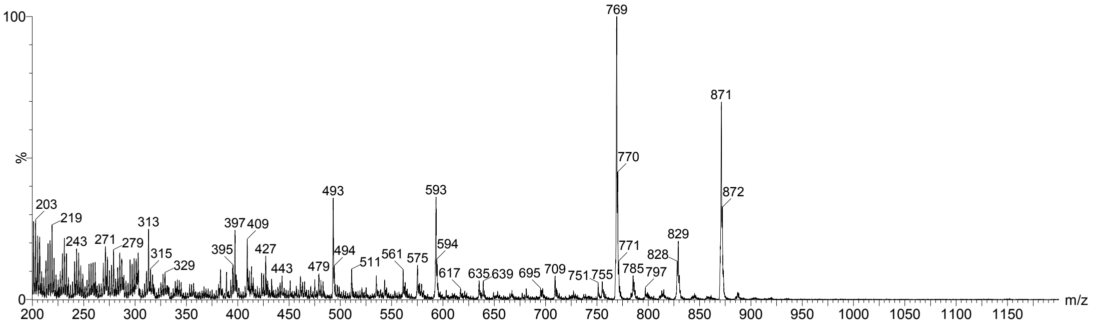

(L) SMS12

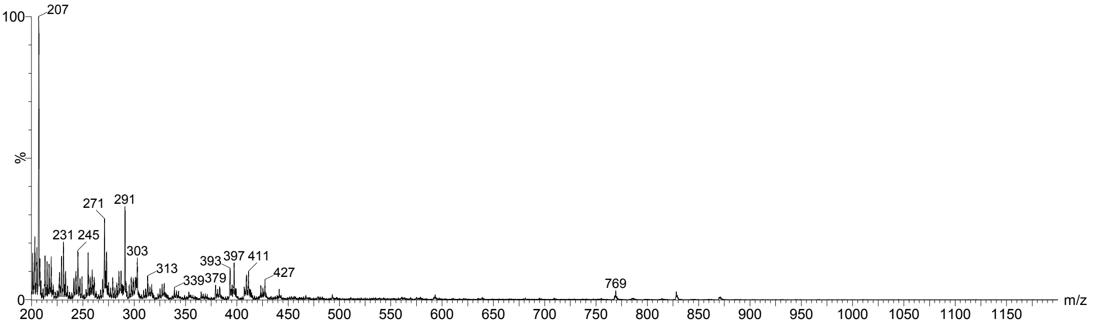

(M) SMS13

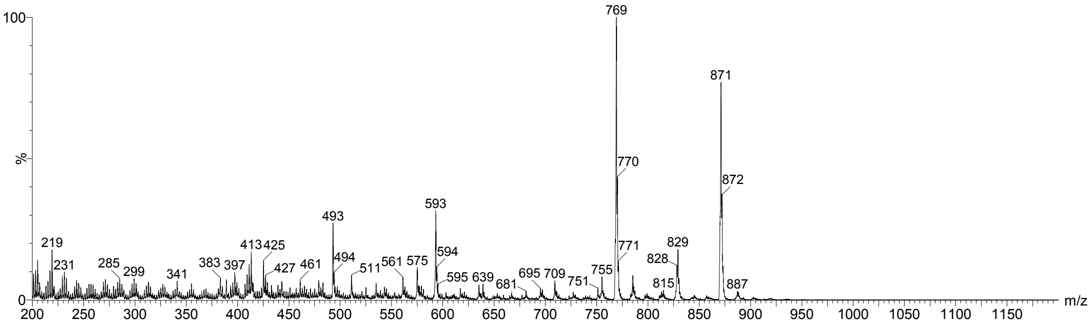

(N) SMS14

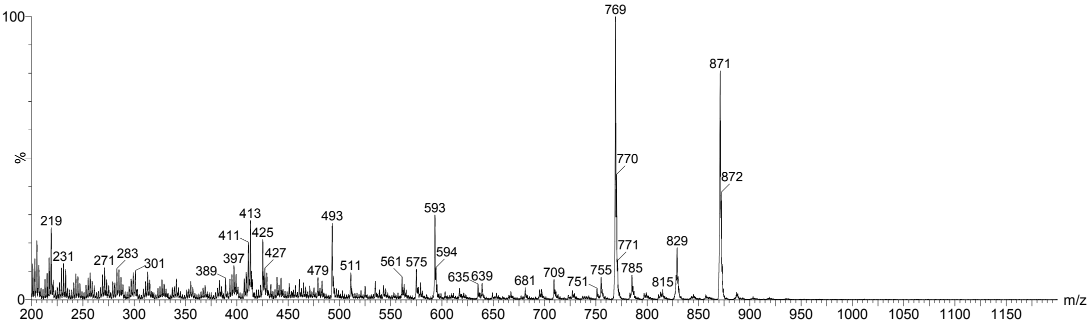

(O) SMS15

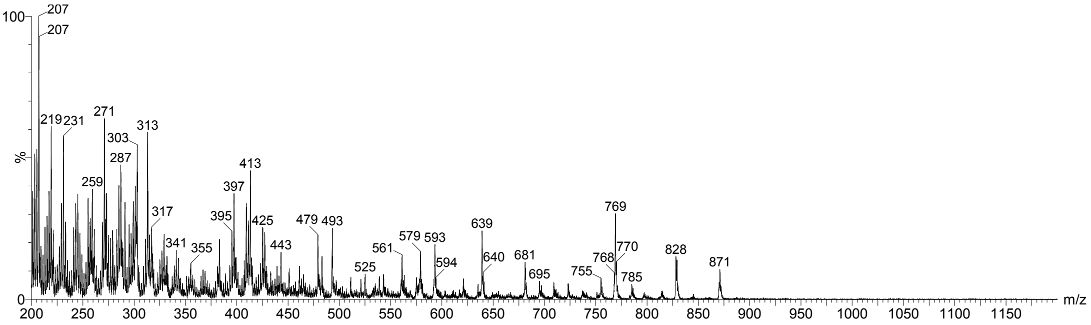

(P) SMS16

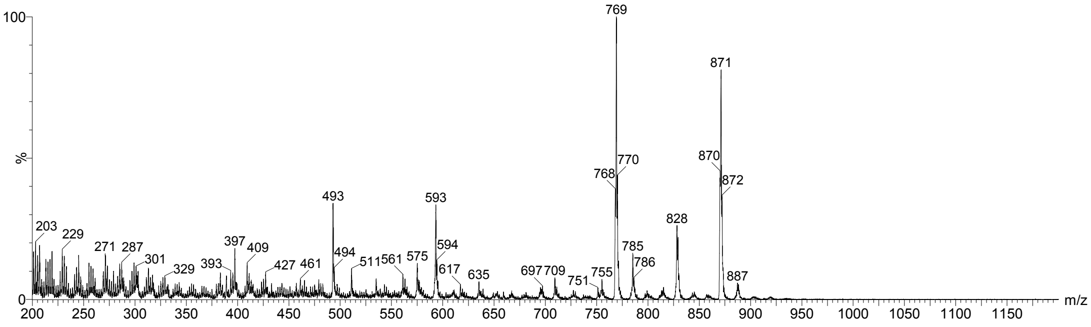

Figure S5. Full scan mass spectra of representative blank samples.

(A) BLANK1

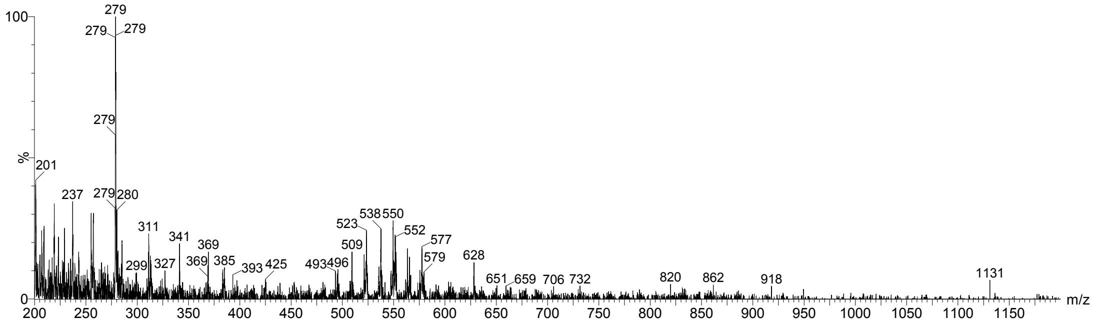

(B) BLANK2

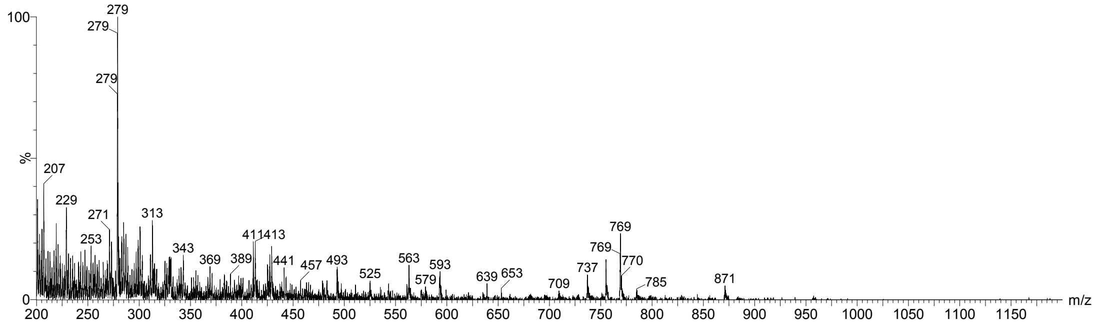

Figure S6. Full scan mass spectra of representative pull samples.

(A) POOL1

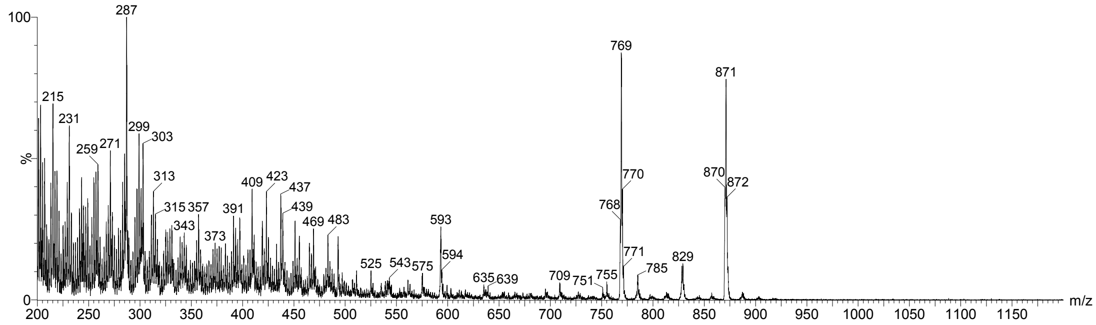

(B) POOL2

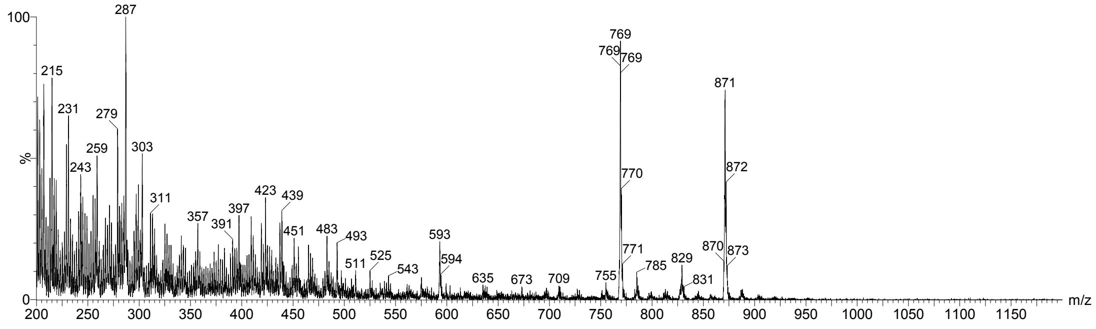

Figure S7. Example of LiveID software recognizing the Andiroba sample in real time.

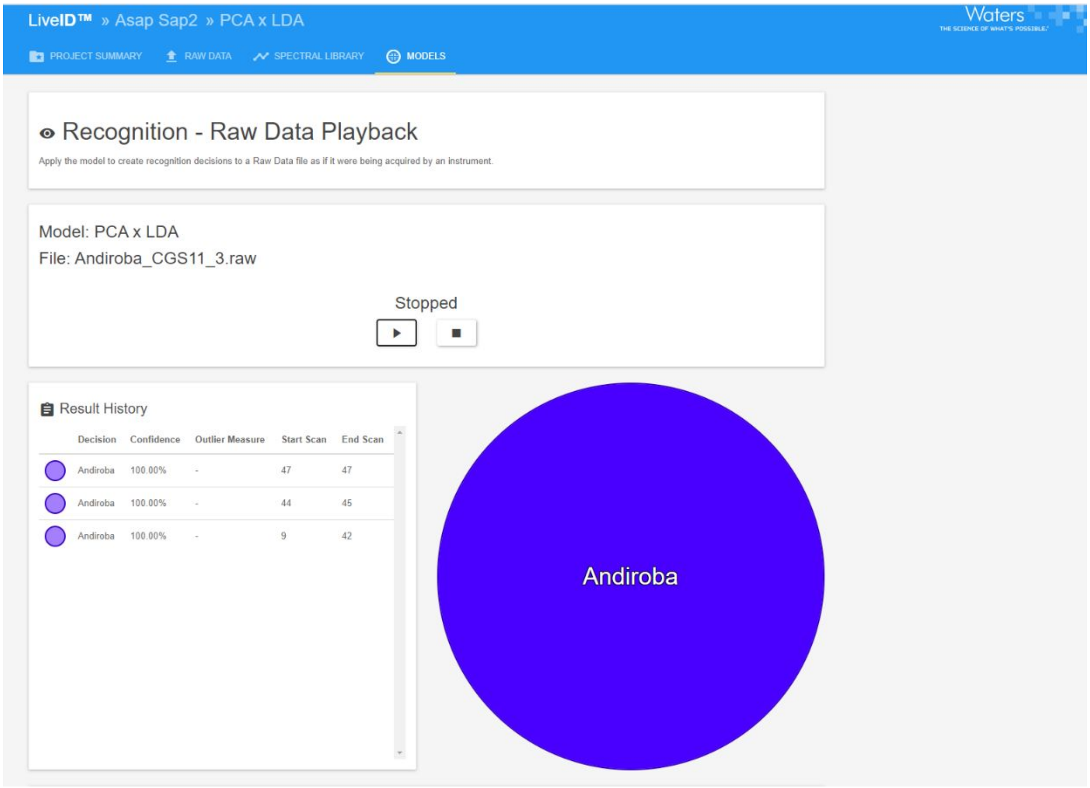

Supplement: Supplementary file 1 [file ao5c13161_si_001.pdf]
